# MXene-modified BiVO_4_/FePO_4_ photoanodes for solar oxygen evolution

**DOI:** 10.1039/d6na00469e

**Published:** 2026-08-18

**Authors:** Micaela Pozzati, Chiara Deriu, Paola Ragonese, Francesco Lamberti, Mattia Cattelan, Veronika Zahorodna, Roberto Altieri, Yana Ihnatenko, Serhii Dukhnovskiy, Ivan Baginskiy, Oleksiy Gogotsi, Isabella Poli, Laura Fabris, Teresa Gatti, Mengjiao Wang

**Affiliations:** a Department of Applied Science and Technology, Politecnico di Torino C.so Duca degli Abruzzi 24 10129 Torino Italy; b Center for Sustainable Future Technologies, Istituto Italiano di Tecnologia Via Livorno 60 10144 Torino Italy; c Dipartimento di Scienze Chimiche, Università degli Studi di Padova Via Marzolo, 1 35131 Padova Italy; d Materials Research Centre 3 Krzhizhanovskogo Str. 03142 Kyiv Ukraine; e Y-Carbon Ltd 18 Bohdana Havrylyshyna st Kyiv Ukraine; f Center for Materials Research, Justus-Liebig University Giessen Heinrich-Buff-Ring 16-17, 35392 Giessen Germany teresa.gatti@polito.it mengjiao.wang@polito.it

## Abstract

Photoelectrochemical reactions in aqueous systems represent a promising route for solar fuel production; however, their efficiency is often constrained by the sluggish kinetics of the anodic oxygen evolution reaction (OER). BiVO_4_ is one of the most promising photoanode materials and has demonstrated the ability to efficiently convert light into electrical and then chemical energy. However, the conversion efficiency of pure BiVO_4_ remains insufficient. In this work, BiVO_4_ photoanodes are first modified with FePO_4_ using a simple Autodrop process and subsequently integrated with Ti_3_C_2_T_*x*_ MXene overlayers deposited *via* automated spray coating. The thus modified photoanodes exhibit a superior performance thanks to the synergistic interaction between the FePO_4_ and MXene layers, which promotes the movement of electrons and efficient charge separation, thus suppressing surface recombination in BiVO_4_. Meanwhile, both the FePO_4_ and MXene layers can act as hole-transport channels and co-catalytic interfaces, facilitating interfacial charge transfer and accelerating OER kinetics. Importantly, achieving an optimal balance between MXene coverage and FePO_4_ exposure is critical to maximizing catalytic activity within the BiVO_4_/FePO_4_/MXene heterostructure. By optimizing the MXene loading, the modified BiVO_4_/FePO_4_ photoelectrode exhibits an approximately 50% improvement in photocurrent performance. Overall, this work presents a simple and effective strategy for fabricating high-performance photoanodes with potential for scalable production. The proposed heterojunction design provides a promising pathway toward the development of efficient and stable photoanodes for solar fuel applications.

## Abstract 2

BiVO_4_ photoanodes integrated with FePO_4_ and Ti_3_C_2_T_*x*_ MXene can achieve more than 50% enhanced photocurrent performance by promoting charge separation, suppressing recombination, and accelerating OER kinetics through synergistic interfacial effects.

## Graphical abstract

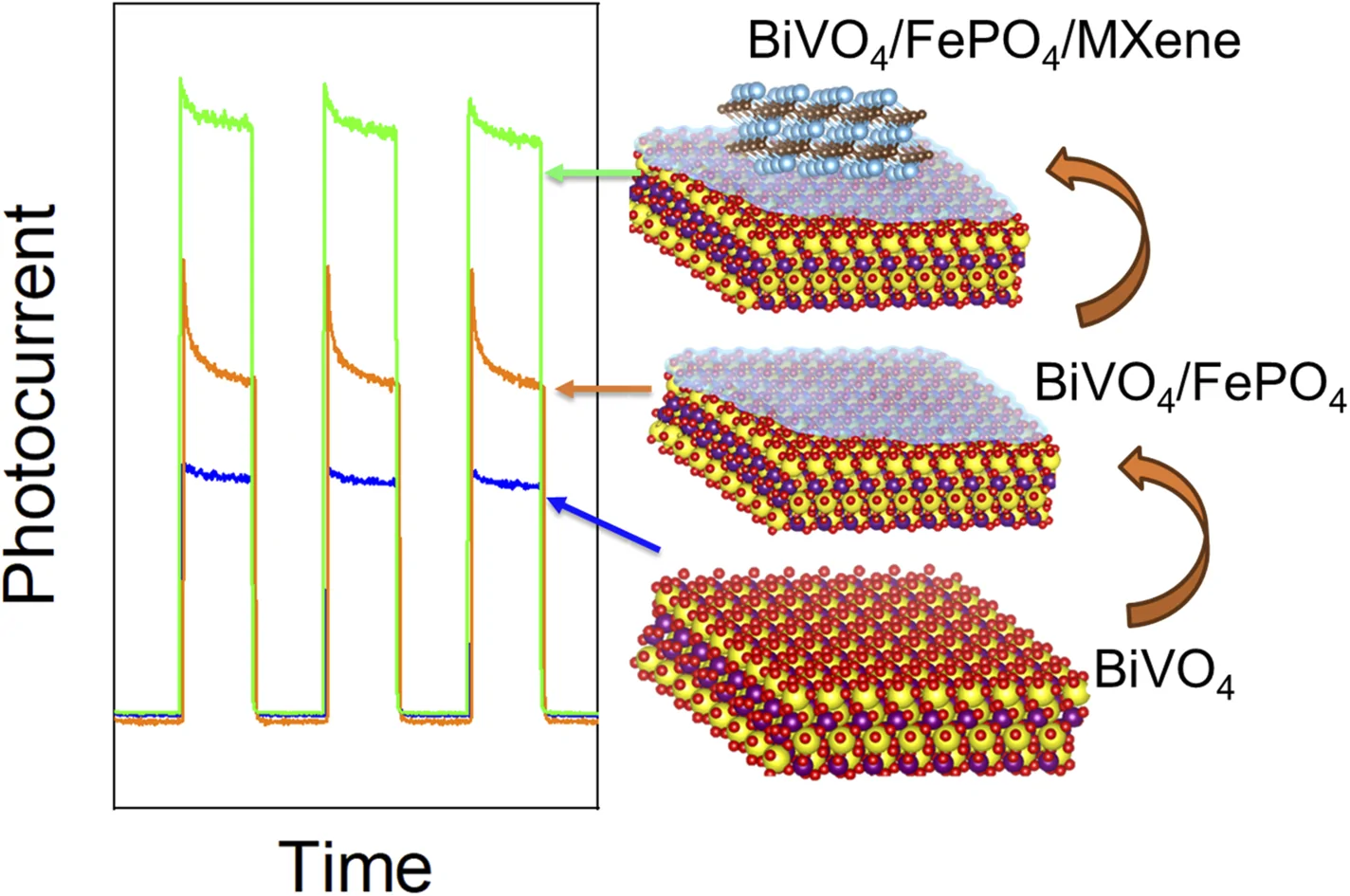

## 1 Introduction

Photoelectrochemical (PEC) reactions offer a promising approach for the sustainable and environmentally friendly conversion of solar and electric energy into renewable fuels.^1,2^ However, the overall efficiency of aqueous PEC systems is severely limited by the intrinsically sluggish kinetics of the anodic oxygen evolution reaction (OER). This bottleneck has motivated extensive research into the development of efficient and stable photoanode materials.^3^ Among the various candidates, BiVO_4_ has emerged as one of the most extensively studied photoanodes for the OER, owing to its good chemical stability in mild pH aqueous solution, optimal band gap (2.4–2.6 eV) for solar light absorption, and a suitable valence band position for the OER, allowing a theoretical photocurrent density of approximately 7.5 mA cm^−2^ to be reached.^4–7^ Despite these advantages, the practical performance of BiVO_4_ remains far below its theoretical limit due to several inherent drawbacks, including poor charge transport properties, severe surface recombination and photo-corrosion under operating conditions.^8^ Therefore, surface and interface engineering strategies are often required to improve charge separation and interfacial charge transfer in BiVO_4_-based photoanodes. To address these limitations, a variety of co-catalysts and surface modifiers have been explored, such as Al_2_O_3_, CoPi, Co_3_O_4_, NiB alloys, and FeOOH.^4,9–12^ Among these surface modifiers, Fe-based compounds are particularly attractive due to the earth-abundant and non-critical nature of iron, as well as its well established role as an efficient active site for the OER. Furthermore, Fe-based layers provide surface passivation, mitigating surface defect states that otherwise would act as recombination centres.^4,13–15^ Materials like FePO_4_ can also function as a hole-transport layer, improving the effective hole transfer.^16^

Recently, two-dimensional conductive materials such as MXenes have emerged as promising interfacial modifiers for semiconductor photoelectrodes due to their high electrical conductivity and large surface area. 2D MXene materials, which are transition metal carbides, nitrides, or carbonitrides, have emerged as promising hole-transport and co-catalytic materials for PEC applications.^17^ MXenes exhibit a unique combination of properties such as high electrical conductivity, hydrophilicity, tunable surface chemistry and high electronegativity, making them attractive for electrochemical and photoelectrochemical systems.^18^ Among them, Ti_3_C_2_T_*x*_, typically derived from the etching of the MAX phase Ti_3_AlC_2_, exhibits high surface reactivity due to abundant surface terminations such as –OH and –F groups, which facilitate interfacial interactions and functionalization.^19,20^ As a result, MXenes have increasingly been explored as co-catalysts in PEC water splitting.^21–23^ In the context of the OER, BiVO_4_/MXene heterostructures have demonstrated enhanced activity and stability through improved charge transfer and suppressed recombination.^24,25^ For instance, Jahangir *et al.* reported a Ti_3_C_2_T_*x*_/BiVO_4_ heterojunction achieving a photocurrent density of 4.72 mA cm^−2^,^17^ which is 1.5 times higher than the photocurrent of pure BiVO_4_, and Cheng *et al.* achieved 5.13 mA cm^−2^ using an alkaline-etched BiVO_4_/MXene system.^26^ In these systems, MXenes act as hole-extraction layers and conductive bridges, reducing series resistance, suppressing charge recombination, and accelerating interfacial charge transfer.^27^ Although these studies demonstrate the promise of MXene-functionalized BiVO_4_ photoanodes, most reported fabrication approaches remain limited to laboratory-scale methods, restricting their potential for large-scale production.^28,29^

In the practical development of modified BiVO_4_ photoanodes, material optimization alone is insufficient; scalability and compatibility with industrial manufacturing should be considered equally critical. Moreover, thin-film fabrication techniques play a decisive role in tailoring the optical and electronic properties of photoanodes, thereby strongly influencing PEC performance. Established methods such as electrodeposition and spray pyrolysis have been widely employed for the fabrication of BiVO_4_-based photoanodes, offering good control over film morphology and enabling significant performance improvements in modified architectures. Recent studies have highlighted their effectiveness in producing high-quality photoactive layers and in integrating cocatalysts or surface modifiers for enhanced PEC activity.^30–33^ However, these techniques may require precise process control, elevated processing temperatures, or multiple post-treatment steps, which can complicate large-scale implementation, particularly for multilayered heterostructures. Conventional synthesis routes for BiVO_4_-based heterostructures, such as hydrothermal methods, are often limited in scalability and reproducibility.^29,34,35^ In contrast, the Autodrop technique, derived from the successive ionic layer adsorption and reaction (SILAR) process, offers a scalable and highly controllable approach for fabricating uniform BiVO_4_-based photoanodes.^36,37^ Our previous work has demonstrated the successful preparation of BiVO_4_/FePO_4_ photoanodes using the Autodrop method.^16,38^ Additionally, automated spray coating has been introduced as an efficient strategy for depositing Ti_3_C_2_T_*x*_ MXene films. This technique enables superior film uniformity, reduced electrical resistance, faster processing, and lower MXene consumption, making it particularly compatible for scalable electrode fabrication.^39,40^ In addition to improving photoelectrode performance, the potential for scalable and controllable deposition methods remains an important aspect for practical implementation of PEC materials.

Building on these advances, combining the Autodrop process with automated spray coating offers a promising route to fabricate BiVO_4_/FePO_4_/MXene photoanodes with the possibility of scaling up. Here, we explore the combined use of an FePO_4_ interlayer and MXene nanosheets to create complementary charge-transfer pathways within the photoanode architecture. In this work, BiVO_4_/FePO_4_ photoanodes were modified with Ti_3_C_2_T_*x*_ MXene nanosheets using an automated spray-coating process. BiVO_4_/FePO_4_ photoanodes were first prepared *via* the Autodrop method, as previously reported by our group,^16,38^ followed by the deposition of exfoliated Ti_3_C_2_T_*x*_ MXene layers using automated spray coating with 1 mg mL^−1^ Ti_3_C_2_T_*x*_ MXene colloidal solution (Fig. 1). By systematically varying the MXene loading, the influence of MXene film thickness on PEC performance is investigated. The optimized BiVO_4_/FePO_4_/MXene photoanode exhibits significantly enhanced PEC water oxidation performance, arising from improved bulk charge separation at the BiVO_4_/FePO_4_ junction and efficient charge transport and co-catalytic OER activity provided by the highly conductive Ti_3_C_2_T_*x*_ MXene. Furthermore, the MXene overlayer enhances photoanode stability under PEC conditions by facilitating continuous hole extraction from the BiVO_4_ semiconductor and preventing its degradation. This work demonstrates a viable strategy for the production of stable and high-performance BiVO_4_/FePO_4_/MXene photoanodes for PEC OER applications.

**Fig. 1:**
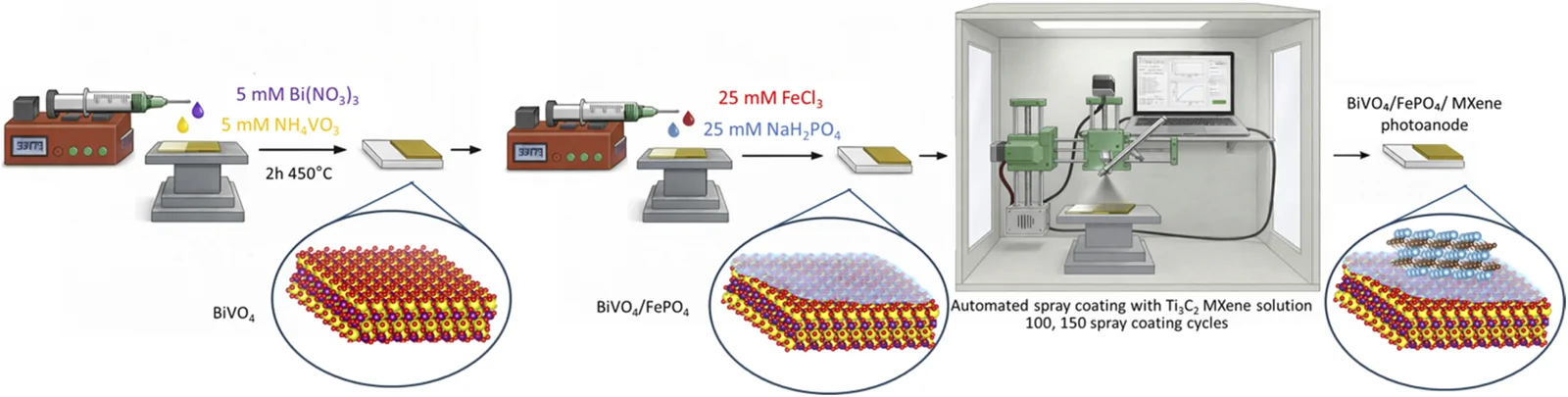
Schematic representation of the Autodrop-spray coating processes used in this work to obtain BiVO_4_/FePO_4_/MXene photoanodes.

## 2 Experimental section

### 2.1 Materials

Bismuth(iii) nitrate pentahydrate (Bi(NO_3_)_3_·5H_2_O), ammonium vanadate (NH_4_VO_3_), sodium dihydrogen phosphate (NaH_2_PO_4_), and iron chloride (FeCl_3_) were acquired from Thermo Fisher. FTO-coated glass substrates were purchased from Sigma-Aldrich. Titanium aluminium carbide (Ti_3_AlC_2_) was obtained from Materials Research Center Ltd. Hydrofluoric acid (HF) was purchased from Honeywell, and hydrochloric acid (HCl) and lithium fluoride (LiF) were acquired from Roth.

### 2.2 Preparation of photoanodes

#### 2.2.1 Autodrop of BiVO_4_ and FePO_4_ layers

The photoanodes were prepared by a procedure already published in our recent work with proof of reproducibility.^16^ BiVO_4_ was synthesized by dispensing a precursor solution containing 5 mM NH_4_VO_3_ and 5 mM Bi(NO_3_)_3_·5H_2_O onto an FTO glass substrate using a KLM spin-coater SCV-10 (Fig. 1). The deposition was carried out using a syringe pump at a dispensing rate of 100 µL min^−1^ and a spinning speed of 40 rps, with a total deposited volume of 2 mL. After deposition, the substrate was annealed at 450 °C for 2 h using a heating ramp of 10 °C min^−1^. FePO_4_ was prepared by the Autodrop method as well, using 25 mM FeCl_3_ and 25 mM NaH_2_PO_4_ as precursor solutions. The deposition parameters were a dispensing rate of 100 µL min^−1^, a spinning speed of 40 rps, and a total deposited volume of 0.4 mL. The obtained films were subsequently annealed at 450 °C for 2 h with a heating ramp of 10 °C min^−1^. The substrate area coated with the active material was 1 × 1 cm^2^.

#### 2.2.2 Exfoliation of MXene and automatic spray-coating of MXene layers

The procedure for MXene flake synthesis followed the one from an already published study.^41^ Ti_3_C_2_T_*x*_ MXene was prepared by selective etching of Ti_3_AlC_2_ in HF formed *in situ*, followed by repeated washing with Milli-Q water and centrifugation to remove residual acid and obtain multilayered Ti_3_C_2_T_*x*_. Single- or few-layer Ti_3_C_2_T_*x*_ MXene was then prepared using a minimally intensive layer delamination (MILD) procedure, in which Ti_3_AlC_2_ was etched in a LiF/HCl solution and the resulting suspension was washed and centrifuged until a dark supernatant containing delaminated MXene flakes was obtained. Collected supernatant was centrifuged at 3000 rcf for 20 minutes to sediment Ti_3_C_2_T_*x*_. The collected sediment was washed several times to remove residual acid and LiF. The obtained Ti_3_C_2_T_*x*_ MXene water based sediment was diluted with DI-water to form a 1 mg mL^−1^ Ti_3_C_2_T_*x*_ suspension that was later on used for the automated spray coating procedure to deposit MXene flakes on top of the BiVO_4_ and BiVO_4_/FePO_4_ photoanodes. Ti_3_C_2_T_*x*_ MXene layers were deposited *via* cycles of short exposure of BiVO_4_/FePO_4_ photoanodes to an aerosol of a water-based Ti_3_C_2_T_*x*_ colloidal solution. In the automated spray-coating process, one deposition cycle consisted of a slow pass of the spray nozzle across the electrode surface (≈1.5 s), followed by a return pass (≈1.5 s) and a short ambient drying period of about 1 s. Two sets of BiVO_4_/FePO_4_/MXene photoanodes were produced that were exposed to 100 and 150 such cycles. The automated spray-coating setup allows programmable control of deposition parameters and deposition cycles, enabling reproducible coating conditions and providing a practical route toward scalable fabrication of MXene-modified photoelectrodes. Pictures of the automated spray coating system are shown in the SI (Fig. S1).

### 2.3 Materials characterization

Scanning electron microscopy (SEM) images were recorded using a TESCAN SEM with an acceleration voltage of 5 kV, a beam current of 100 pA and a sensor to acquire and analyze the energy-dispersive X-ray spectra (EDS). Diffractogram spectra were acquired on a Malvern PANalytical Empyrean Series-3 diffractometer (Cu Kα radiation, Malvern Panalytical Ltd) at 40 kV and 40 mA. Standard, single-point Raman spectra were acquired at room temperature by employing a Renishaw inVia spectrometer, equipped with a 532 nm laser. The measurements were performed with a 50× objective, with a laser power of 0.1 mW, an integration time of 3 s, and 15 accumulations. Fiber-optic confocal Raman microscopy measurements were performed using a WITec alpha300 Apyron confocal microscope, fitted with a lens-based, 400 mm focal length ultrahigh throughput spectrometer (UHTS) and a Peltier-cooled back-illuminated deep depletion CCD camera for measurements in the near-infrared range (*λ*_exc_ 785 nm). All acquisitions were performed utilizing a 300 g mm^−1^ grid and a Zeiss semi-apochromatic, EC Epiplan-Neofluar 100× objective with a numerical aperture of 0.9, yielding an airy disk laser spot diameter of 1.064 µm for the 785 nm excitation line. Acquisition parameters were optimized to maximize the signal to noise (S/N) ratio while keeping the irradiance in the order of tens of thousands of W cm^−2^,^41^ to avoid laser-induced MXene degradation: 1 s integration time, 5 accumulations, and a power of 0.5 mW at the objective, for an irradiance of 56.2 kW cm^−2^. Mapping was performed over representative areas of the samples, ranging from a minimum of 10 × 12 µm^2^ to a maximum of 30 × 30 µm^2^, with a spatial resolution of 0.5 µm along both *x* and *y* directions. All generated spectra were treated for cosmic ray removal using the embedded filtering function in the WITec Project SIX Plus software; no baseline correction, background removal, or smoothing was performed. Raman images were generated from the acquired spectral maps by integrating each selected band using the peak maximum as the center and the baseline-defined width as the wavenumber limits. Spectral resolution for all measurements at *λ*_exc_ 785 nm is 1.91–1.36 cm^−1^ across the 100 to 2000 cm^−1^ range. X-ray photoelectron spectroscopy (XPS) spectra were acquired in an ultra-high-vacuum (UHV) chamber with a working pressure below 5 × 10^−8^ mbar, using the Al Kα line (*hν* = 1486.7 eV). Quantitative elemental analysis was carried out using sensitivity-factor-corrected peak areas extracted from the high-resolution spectra. Owing to the layered nature of the electrodes and the different information depths associated with the analysed core levels, elemental ratios were considered only as approximate estimates and were not used for rigorous stoichiometric analysis.

UV-vis diffuse reflectance spectra were recorded on the FTO substrate using a Jasco V-770 spectrophotometer, coupled with an integrating sphere. Band gap values were obtained from absorbance spectra using the Tauc plot equation:(*αhν*)^1/*n*^ = *A*(*hν* − *E*_g_)where *α* is the absorption coefficient, *h* is the Planck constant, *ν* is the frequency, *E*_g_ is the bandgap energy, and *n* is ½ for direct band gap materials such as BiVO_4_.^42^

### 2.4 Photoelectrochemical characterization

PEC measurements were conducted with an Autolab PGSTAT204 potentiostat and a Spectral Products ASB-XE-E180 xenon lamp light source, set to 100 mW cm^−2^ using an Oriel PV reference cell system. The substrate was irradiated with AM 1.5G light. The setup used was a three-electrode cell, employing an Ag/AgCl (saturated KCl) reference electrode, a platinum wire counter electrode, and a platinum clip as the working electrode. The electrolyte employed was Na_2_SO_4_ 0.5 M with pH = 7. The samples were front illuminated through a quartz window on an Ossila glass PEC cell. Potentials referenced to the Ag/AgCl were converted to the RHE scale according to the following equation:*E* (V *vs.* RHE) = *E* (V *vs.* Ag/AgCl) + 0.197 V + 0.059 V × pH

LSV curves were acquired at a scan rate of 10 mV s^−1^. EIS was conducted at 1.23 V, 1.4 V and 1.6 V *vs.* RHE under simulated solar light, in a range of frequencies from 100 kHz to 1 Hz. IPCE was calculated using 6 Thorlabs FKBV10 hard-coated Vis bandpass filters with wavelengths from 350 to 600 nm (10 nm fwhm) and the xenon lamp. The IPCE at a specific wavelength (*λ*) was obtained using the following formula:
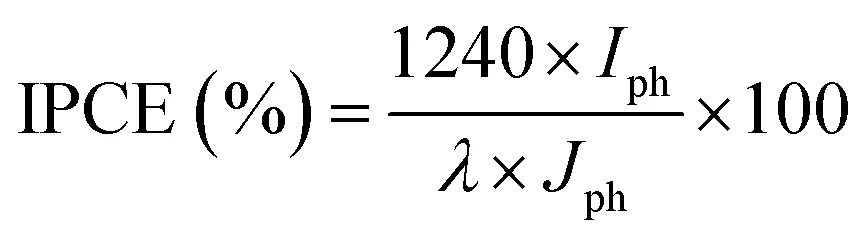
where *I*_ph_ is the photocurrent density, *λ* is the incident wavelength and *J*_ph_ is the incident irradiance, measured thanks to an 843-R Newport optical power meter. Mott–Schottky measurements were performed using a BioLogic SP-150e potentiostat in the dark at 1 kHz. The donor density (*N*_d_) was obtained from the slope d(1/*C*_SC_^2^)/d*V*, and the flat band potential can be obtained from the intercept of the linear fit with the *X* axis (1/*C*_SC_^2^ = 0), both using the formula:
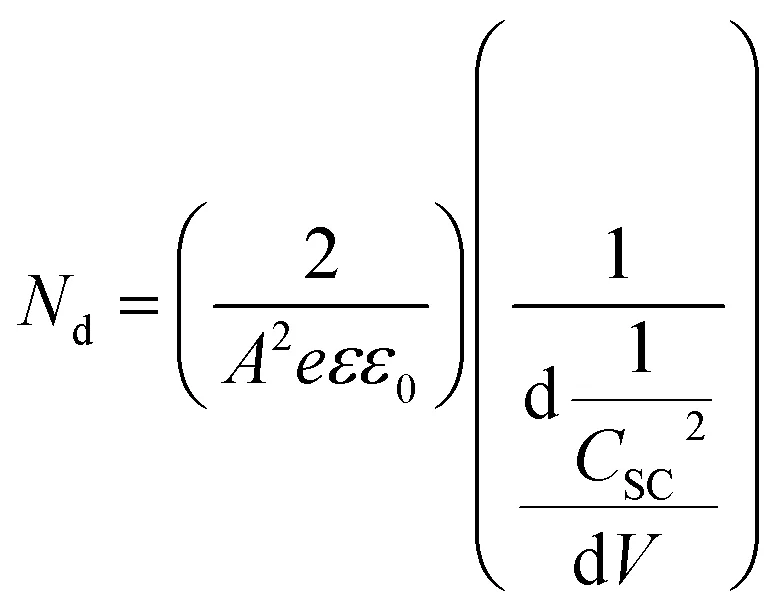
where *A* is the electrode area, *e* is the electron charge, *ε* is the dielectric constant for BiVO_4_ (ref. 36 and 43) and *ε*_0_ is the permittivity in a vacuum.

LSV curves with scavengers are measured in the aqueous electrolyte containing 0.5 M of Na_2_SO_4_ and 0.5 M of Na_2_SO_3_, and Na_2_SO_3_ acts as the hole scavenger. The charge transfer efficiency (*η*_tr_) at 1.23 V *vs.* RHE is calculated using the following equation:
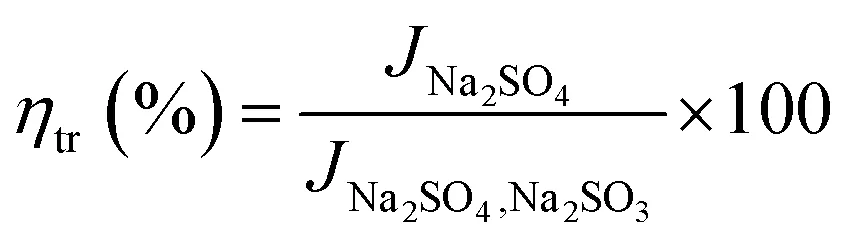
where *J*_Na_2_SO_4__ is the photocurrent density measured in 0.5 M of Na_2_SO_4_ and *J*_Na_2_SO_4_,Na_2_SO_3__ is the photocurrent density measured in an electrolyte containing Na_2_SO_3_.

Applied Bias Photon-to-Current Efficiency (ABPE) was calculated using the following equation:
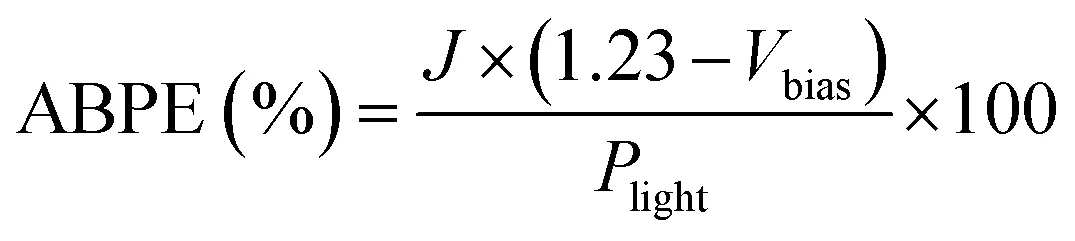
where *J* is the photocurrent density, *V*_bias_ is the applied potential *vs.* RHE (0.8V *vs.* RHE) and *P*_light_ is the light intensity (100 mW cm^−2^).

IMPS data were obtained with an Ivium CompactStat potentiostat coupled with a LED ModuLight (Ivium). The measurements were acquired at 1.23 V *vs.* RHE in Na_2_SO_4_ 0.5 M (pH = 7) with a white LED (*P* = 20 mW cm^−2^). The light intensity was measured using a Si-photodiode positioned at the same location as the sample. The amplitude oscillation was set to 10% of the DC light intensity, with the modulation frequency ranging from 5 kHz to 0.2 Hz. The acquired data are displayed in the complex plot of the dimensionless transfer function defined as
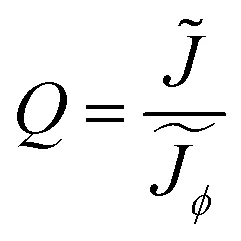
where 
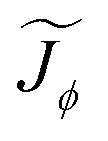
 = *qφ* is the modulated photon flux expressed as current density, with *φ* being the photon flux (s^−1^ cm^−2^) at 550 nm (the maximum wavelength of the emission spectrum of the light) and *q* being the elementary charge. *J̃* is the corresponding modulating photocurrent density (A cm^−2^).

## 3 Results and discussion

### 3.1 Structural and morphological characterization

BiVO_4_ and BiVO_4_/FePO_4_ electrodes were fabricated on fluorine-doped tin oxide (FTO) substrates using an Autodrop setup (see the Experimental section and Fig. 1 for details).^16^ The optimized fabrication parameters for BiVO_4_ and BiVO_4_/FePO_4_ photoanodes were selected based on our group's previous report. Subsequently, MXene layers were deposited onto the electrodes *via* an automated spray coating process using exfoliated MXene nanosheets prepared by the etching and delamination procedures described in the Experimental section. Two MXene loadings were tested by applying 100 and 150 automated spray coating cycles. Accordingly, the MXene-modified electrodes are denoted as BiVO_4_ 100L, BiVO_4_ 150L, BiVO_4_/FePO_4_ 100L, and BiVO_4_/FePO_4_ 150L, where “L” refers to the number of spray-coating layers. The structural properties were analysed with X-ray diffraction (XRD) (Fig. S2). All the samples exhibit identical diffraction features, which can be primarily assigned to the monoclinic scheelite phase of BiVO_4_ (ICSD 100602).^44^ No crystalline FePO_4_ phases are detected, consistent with the amorphous nature of FePO_4_ synthesized *via* the Autodrop process.^16^ For BiVO_4_/FePO_4_ 150L, a broad diffraction peak centered at ∼7.5° is observed and attributed to the (002) reflection of Ti_3_C_2_T_*x*_. This peak is shifted from 9.8° in the Ti_3_AlC_2_ phase (ICSD 153266) due to the etching process, which removes the Al layers and increases the interlayer spacing of the titanium carbide structure. The enlarged interlayer distance, together with the broadening of the (002) peak, indicates the formation of highly exfoliated and ultrathin MXene layers.^45^ No distinct diffraction peaks corresponding to MXene are observed for the BiVO_4_/FePO_4_ 100L sample, which is likely due to the low loading and ultrathin nature of the deposited MXene nanosheets. Nevertheless, the presence of MXene in all MXene-modified samples is further confirmed by the morphological analysis shown in Fig. 2. SEM combined with EDX analysis was employed to investigate surface morphology and elemental distribution. As shown in Fig. 2, all the samples exhibit a heterogeneous surface morphology dominated by fine granular particles characteristic of BiVO_4_ (Fig. 2a and b) and BiVO_4_/FePO_4_ (Fig. 2c and d). The introduction of the thin FePO_4_ layer does not noticeably alter the surface morphology of the electrode. With increasing MXene loading, the granular matrix becomes progressively covered by ultrathin MXene nanosheets, indicating a high degree of exfoliation. In samples coated with 150 spray-coating cycles (Fig. 2b and d), MXene flakes are more clearly visible compared to those with 100 layers (Fig. 2a and c). However, the surface coverage remains non-uniform, leaving partially exposed regions of the underlying BiVO_4_/FePO_4_ matrix. EDX elemental mapping (Fig. 2e–j) reveals the presence of V, Bi, Fe, P, and Ti, confirming the successful deposition of both FePO_4_ and MXene layers on the BiVO_4_ substrate. Notably, the spatial distribution of Ti closely matches the flake-like features observed in the SEM image, further verifying that the thin overlayer corresponds to Ti_3_C_2_T_*x*_ MXene.

**Fig. 2:**
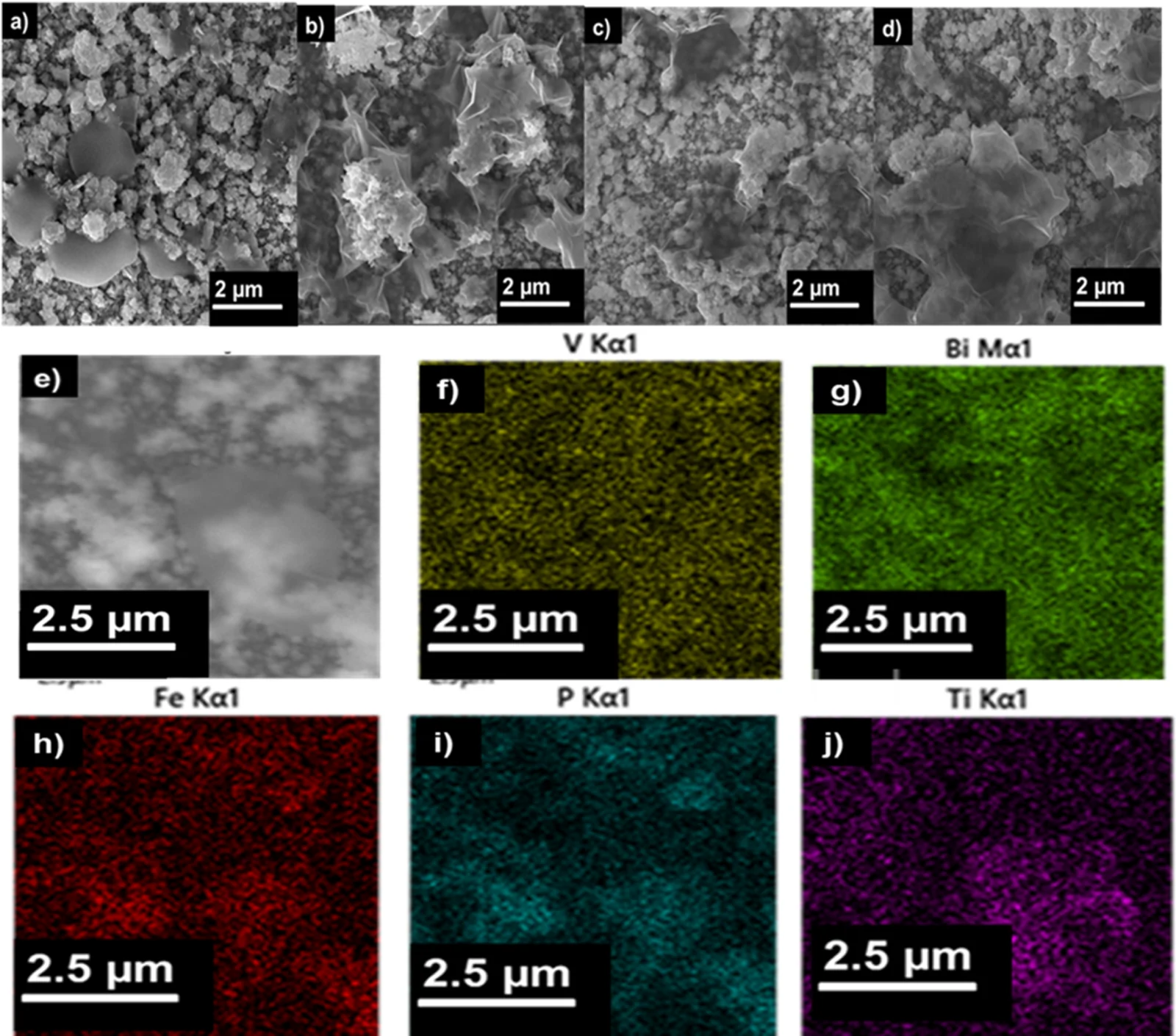
SEM image of (a) BiVO_4_ 100L; (b) BiVO_4_ 150L; (c) BiVO_4_/FePO_4_ 100L; (d) BiVO_4_/FePO_4_ 150L; (e) SEM image; EDX maps of BiVO_4_/FePO_4_ 100L (f) EDX map of V; (g) EDX map of Bi; (h) EDX map of Fe; (i) EDX map of P; (j) EDX map of Ti.

### 3.2 Surface characterization

Standard single-point Raman spectra of the samples are presented in Fig. S3. Using a conventional excitation wavelength for BiVO_4_-based materials (*λ*_exc_ = 532 nm), all the samples display identical Raman features, regardless of their layered composition. These bands are characteristic of monoclinic scheelite-type BiVO_4_.^37^ The peaks at 326 and 366 cm^−1^ are assigned to the antisymmetric and symmetric bending modes of the tetrahedral VO_4_^3−^ units, respectively. Additionally, a shoulder at 710 cm^−1^ and a prominent band at 827 cm^−1^ correspond to the antisymmetric and symmetric V–O stretching vibrations.^46^ As expected under these measurement conditions, amorphous FePO_4_ is not detected. Likewise, no distinct vibrational modes attributable to MXene are observed. These results can be reasonably explained by several factors: (i) the ultrathin nature of the MXene overlayer,^47^ (ii) resonance enhancement of the BiVO_4_ signal due to the excitation wavelength being close to its band gap (Fig. 4),^48^ and (iii) the laser focusing configuration, which results in a penetration depth and focal volume that preferentially probe the underlying BiVO_4_ rather than the superficial ultrathin MXene layers.

**Fig. 3:**
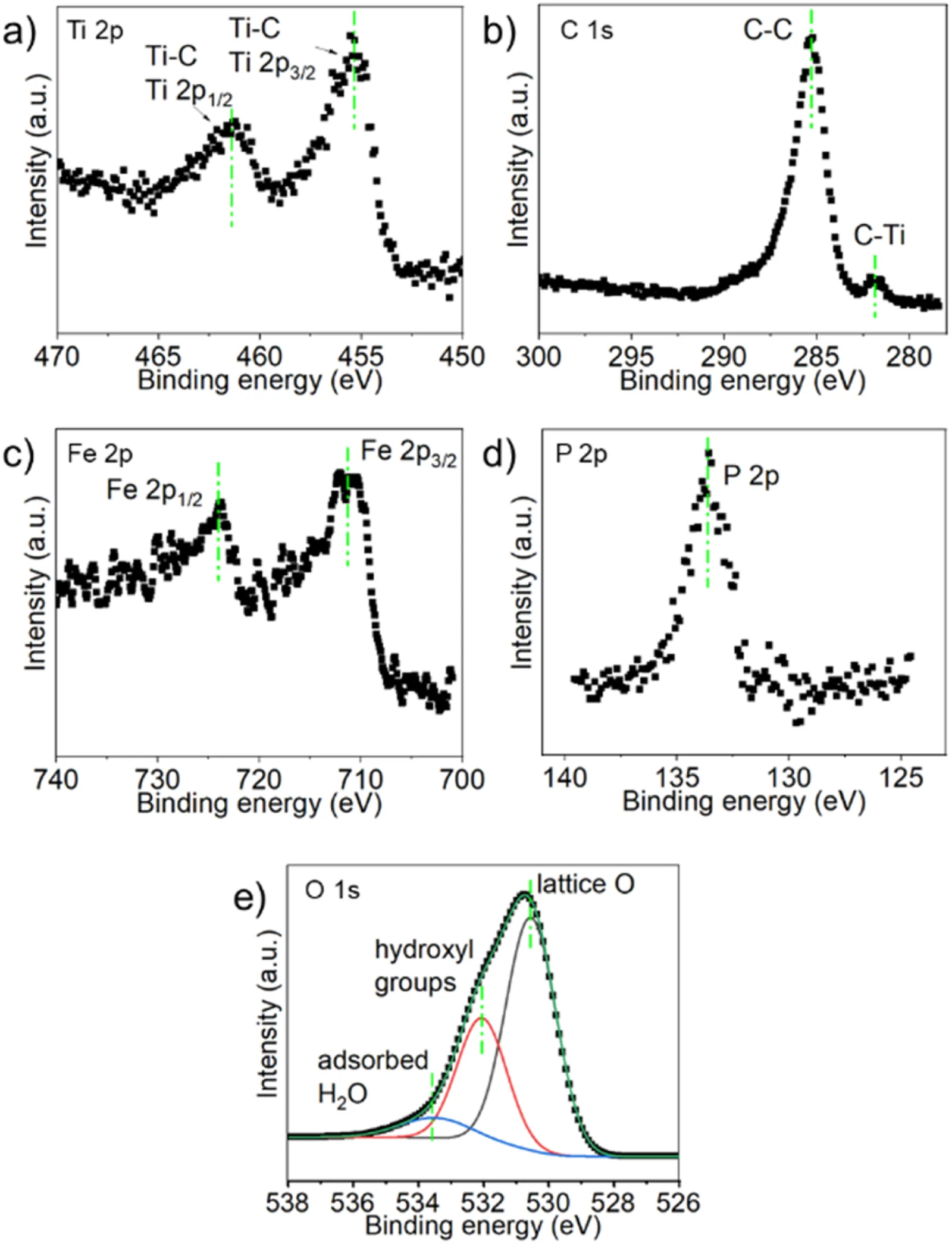
High resolution XPS spectra of BiVO_4_/FePO_4_ 100L: (a) Ti 2p, (b) C 1s, (c) Fe 2p, (d) P 2p and (e) O 1s.

**Fig. 4:**
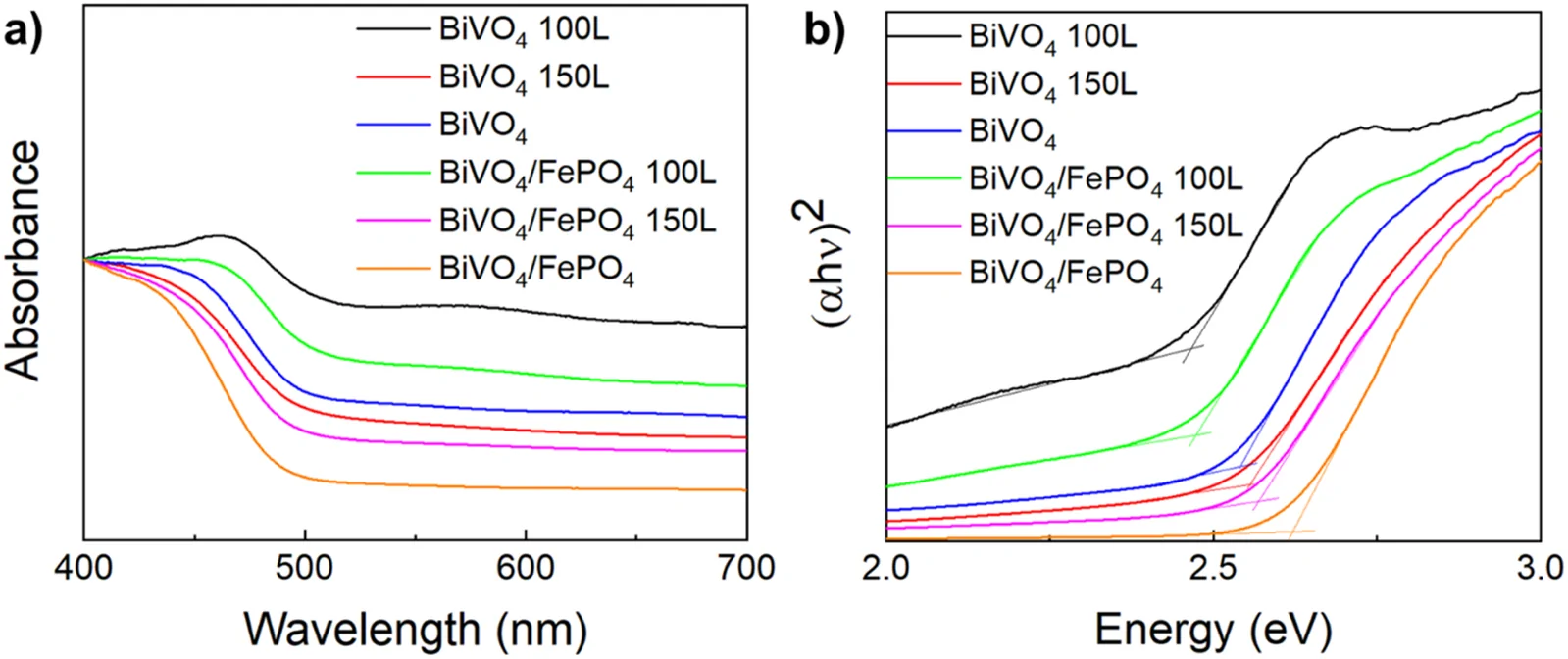
(a) UV-vis absorption spectra and (b) Tauc plots of all the studied photoelectrodes.

To directly assess the surface chemistry of the pristine BiVO_4_/FePO_4_/MXene photoanode, XPS analysis was performed on the representative BiVO_4_/FePO_4_ 100L sample. The survey spectrum shows no detectable Cl, F, Sn, Al, or Li signals, excluding significant residual chloride from FeCl_3_ precursors, detectable residues from MXene etching/delamination processes, and measurable exposure of the underlying FTO substrate (Fig. S4). The Ti 2p and C 1s spectra provide evidence for the presence of Ti_3_C_2_-derived MXene on the electrode.^49^ In particular, the Ti 2p region shows a low-binding-energy contribution at approximately 455.3 eV for Ti 2p_3/2_ and 461.4 eV for Ti 2P_1/2_, which are characteristic peaks of Ti–C bonding in titanium carbide/MXene environments. This assignment is further supported by the C 1s spectrum, where a carbide-related component is observed at approximately 282.0 eV. The simultaneous observation of these Ti–C and C–Ti contributions confirm the presence of TiC-like bonding associated with the MXene phase in the BiVO_4_/FePO_4_ 100L photoanode. Importantly, the preservation of these carbide-related features indicates that the deposition onto the underlying BiVO_4_/FePO_4_ layer does not substantially alter the intrinsic carbide chemistry of the MXene flakes. In addition, the Fe 2p and P 2p regions confirm the presence of Fe-containing phosphate species at the electrode surface. In particular, the Fe 2p line shape is consistent with the intended formation of an FePO_4_ interfacial layer, in agreement with reference FePO_4_ spectra reported in the literature.^50^ The Bi 4f and V 2p signals are strongly attenuated, consistent with the presence of superficial FePO_4_/MXene-derived overlayers within the XPS probing depth (Fig. S4). The O 1s spectrum can be deconvoluted into three main components associated with lattice oxygen (530.6 eV), hydroxyl groups (532.1 eV), and adsorbed water species (533.5 eV) (Fig. 3e).^51^ The spectra are dominated by lattice oxygen and hydroxyl-related contributions, indicating no major restructuring of the oxide/phosphate surface.

### 3.3 Optical characterization and band gap analysis

UV-vis diffuse reflectance spectroscopy (DRS) equipped with an integrating sphere was employed to investigate the optical properties of the samples. The optical band gaps were determined from the UV-vis spectra using Tauc plots assuming a direct allowed transition (Fig. 4). Pristine BiVO_4_ exhibits an absorption edge at approximately 490 nm, corresponding to a direct band gap of 2.54 eV, in good agreement with previous reports.^52^ Upon modification with amorphous FePO_4_ and MXene, the band gap values remain within a range of 2.45–2.60 eV, indicating that these surface modifications do not significantly alter the intrinsic electronic structure of BiVO_4_. This observation confirms that FePO_4_ and MXene are predominantly superficial layers and induce only subtle changes in the fundamental band structure. Nevertheless, slight variations among the samples can be observed. The absorption edge of BiVO_4_/FePO_4_ shows a minor blueshift accompanied by a small increase in the apparent band gap, suggesting limited electronic interaction between BiVO_4_ and the amorphous FePO_4_ overlayer.^53^ Overall, the absorption spectra remain dominated by the characteristic BiVO_4_ absorption edge in the visible region. In contrast, the introduction of thin MXene coatings (100 layers) results in a slight redshift of the absorption edge and a reduced apparent band gap, along with enhanced visible-light absorption and a broadened photoresponse region. This behavior is attributed to strong interfacial electronic coupling at the BiVO_4_/MXene interface, which promotes charge-transfer-related optical transitions and introduces additional electronic states near the band edges.^35^ Because the MXene layer remains thin and partially discontinuous at this loading, these interfacial effects are maximized without significantly obscuring the intrinsic absorption characteristics of BiVO_4_, as supported by the SEM observations (Fig. 2). However, further increasing the MXene thickness to 150 spray coating layers does not lead to a continued decrease in the band gap. Instead, the apparent band gap shifts back toward that of pristine BiVO_4_. This can be explained by the formation of thicker MXene overlayers, where upper MXene sheets partially screen the interfacial interaction between the underlying MXene layer and the BiVO_4_ (or BiVO_4_/FePO_4_) surface. As a result, the electronic coupling becomes less effective, diminishing charge transfer contributions and allowing the intrinsic BiVO_4_ optical response to dominate.^28^

### 3.4 Band edge assessment (Mott–Schottky plot)

Electrochemical impedance spectroscopy was employed to evaluate the flat-band potential (*E*_fb_) and donor density (*N*_d_) of the photoanodes. The Mott–Schottky plots (Fig. 5) show positive slopes for all samples, confirming their characteristic n-type behavior. The flat-band potential was determined by extrapolating the linear portion of the 1/*C*^2^*versus* potential plot to the potential axis. Physically, *E*_fb_ corresponds to the applied potential at which band bending at the semiconductor/electrolyte interface disappears, meaning that the energy bands are flat and no space-charge region is present. As shown in Fig. 5, BiVO_4_/FePO_4_ 100L exhibits the most positive flat-band potential (0.25 V *vs.* RHE) among all samples, indicating that the conduction band edge shifts toward more positive potentials. While the deposition of MXene alone does not significantly alter the position of *E*_fb_, the introduction of the FePO_4_ layer clearly shifts *E*_fb_ toward more positive values (Table 1), highlighting its influence on interfacial energetics. The slopes of the MXene-modified samples remain comparable to those of pristine BiVO_4_ and BiVO_4_/FePO_4_, suggesting that MXene primarily affects surface energetics rather than substantially modifying the bulk carrier density.^16^ The donor densities were calculated using the Mott–Schottky equation (see the Experimental section). The incorporation of MXene leads to a slight increase in donor density from 7.43 × 10^17^ cm^−3^ for BiVO_4_/FePO_4_ to 7.51 × 10^17^ cm^−3^ for BiVO_4_/FePO_4_ 100L, indicating a marginal enhancement in charge carrier concentration. However, excessive MXene loading (150 layers) results in a reduced donor density and a more negative flat-band potential (0.14 V *vs.* RHE for BiVO_4_/FePO_4_ 150L), which may be attributed to electronic shielding effects caused by the thicker MXene overlayer. Additionally, FePO_4_ incorporation itself contributes to an increase in donor density, as BiVO_4_/FePO_4_ exhibits a higher *N*_d_ compared to pristine BiVO_4_.

**Fig. 5:**
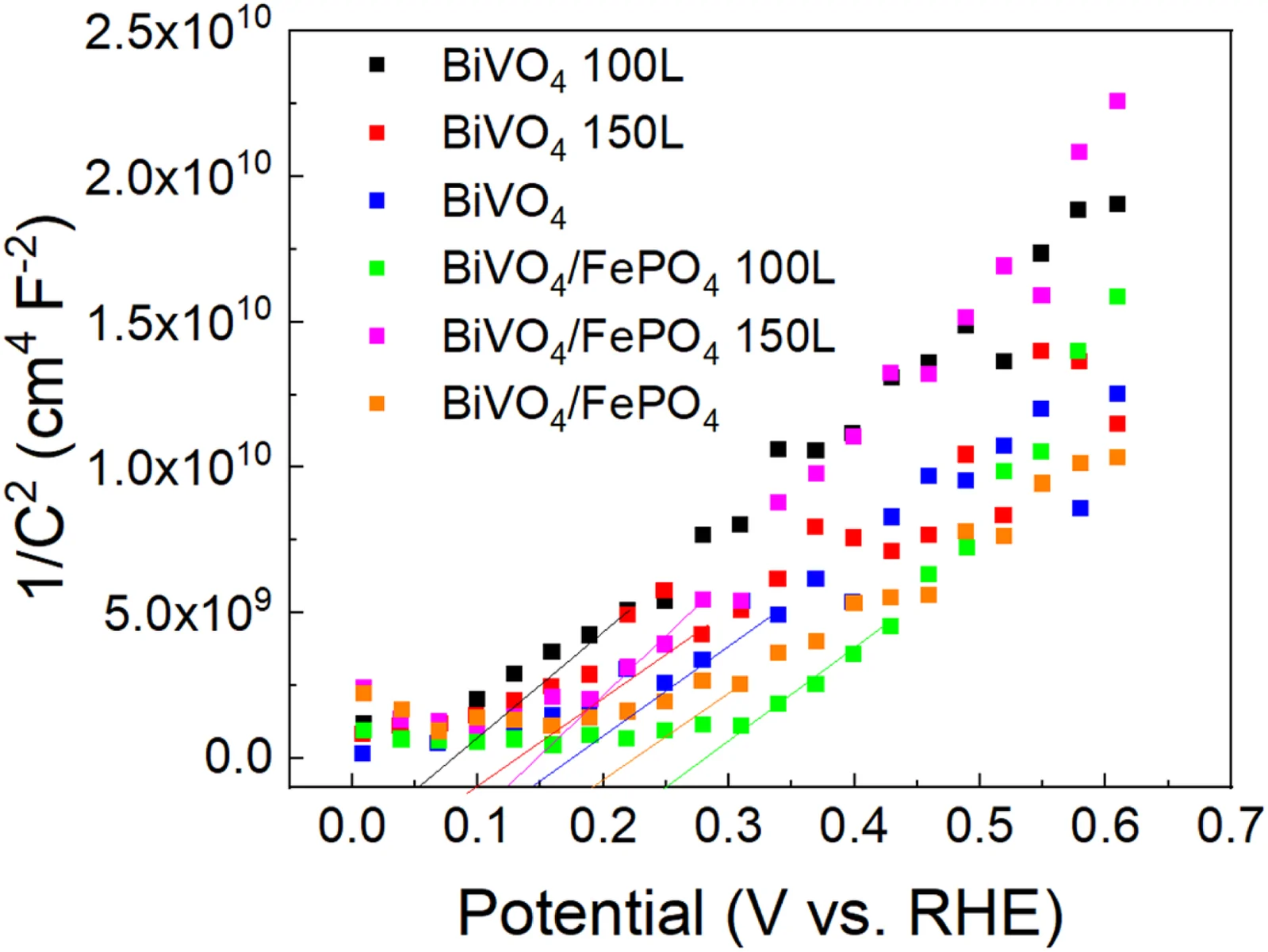
Mott–Schottky plot of all the studied photoelectrodes.

**Table 1:** Values of slope, *E*_fb_, donor density *N*_d_, energy of conductive band *E*_CB_, energy of valence band *E*_VB_ (obtained from Mott–Schottky plots)^54^ and band gap *E*_g_ (obtained from Tauc plots) of all the studied photoelectrodes

Sample	BiVO_4_ 100L	BiVO_4_ 150L	BiVO_4_	BiVO_4_/FePO_4_ 100L	BiVO_4_/FePO_4_ 150L	BiVO_4_/FePO_4_
Slope	3.69 × 10^10^	3.00 × 10^10^	2.90 × 10^10^	2.77 × 10^10^	4.81 × 10^10^	2.80 × 10^10^
*E*_fb_ (V *vs.* RHE)	0.05	0.10	0.12	0.24	0.14	0.19
*N*_d_ (cm^−3^)	5.60 × 10^17^	6.90 × 10^17^	7.17 × 10^17^	7.51 × 10^17^	4.33 × 10^17^	7.43 × 10^17^
*E*_CB_ (V *vs.* RHE)	−0.02	0.03	0.05	0.17	0.06	0.12
*E*_VB_ (V *vs.* RHE)	2.45	2.58	2.59	2.64	2.63	2.74
*E*_g_ (eV)	2.47	2.55	2.54	2.47	2.57	2.62

### 3.5 Photoelectrochemical characterization

#### 3.5.1 PEC OER

The photoelectrochemical performances were assessed by studying the OER for all the prepared samples. All measurements were performed in 0.5 M Na_2_SO_4_ aqueous electrolyte (pH 7) under simulated AM 1.5G solar irradiation. The corresponding linear sweep voltammetry (LSV) curves are shown in Fig. 6. Negligible dark currents are observed for all photoanodes, indicating the absence of significant electrochemical reactions in the dark. The onset potential (at which a positive current is measured) for the PEC OER remains at approximately 0.45 V *vs.* RHE for all samples, suggesting that the fundamental reaction mechanism is not markedly altered by the surface modifications. Nevertheless, the photocurrent density is strongly influenced by the incorporation of FePO_4_ and MXene. The introduction of the FePO_4_ layer enhances the photocurrent compared to pristine BiVO_4_, as evidenced by the higher current densities across the applied potential range. The beneficial effect of MXene is observed only when a thin layer (100 layers) is applied, as shown in Fig. 6a and d. In contrast, samples with thicker MXene coatings (150 layers) exhibit reduced photocurrent densities (Fig. 6b and e), indicating that excessive MXene loading negatively affects charge transport or increases recombination. The best performance is achieved by the BiVO_4_/FePO_4_ 100L photoanode, which delivers a photocurrent density of 2.3 mA cm^−2^ at 1.23 V *vs.* RHE. This value represents nearly a twofold enhancement compared to the BiVO_4_/FePO_4_ electrode without MXene (1.3 mA cm^−2^ at 1.23 V *vs.* RHE), highlighting the synergistic effect of optimized FePO_4_ and MXene surface modification. The improved photoelectrochemical performance can therefore be attributed to a synergistic effect between the FePO_4_ interlayer and the MXene overlayer. The FePO_4_ layer likely facilitates hole transfer and passivates surface states of BiVO_4_, while the highly conductive MXene nanosheets provide efficient pathways for electron extraction. Such complementary charge-transfer pathways help suppress interfacial recombination and promote directional charge transport across the photoanode–electrolyte interface. This cooperative interfacial design plays a key role in enhancing the PEC performance. A comparison with representative BiVO_4_-based photoanodes reported in the literature is provided in Table S1.^4,17,55–57^ Although the PEC performance reported here does not yet match the current state-of-the-art PEC performance, the significance of this work lies in demonstrating a scalable proof-of-concept for constructing BiVO_4_/FePO_4_/MXene heterostructured photoanodes using the combined Autodrop and automated dip-coating strategy. This fabrication approach provides a versatile platform for future optimization of both material composition and device performance.

**Fig. 6:**
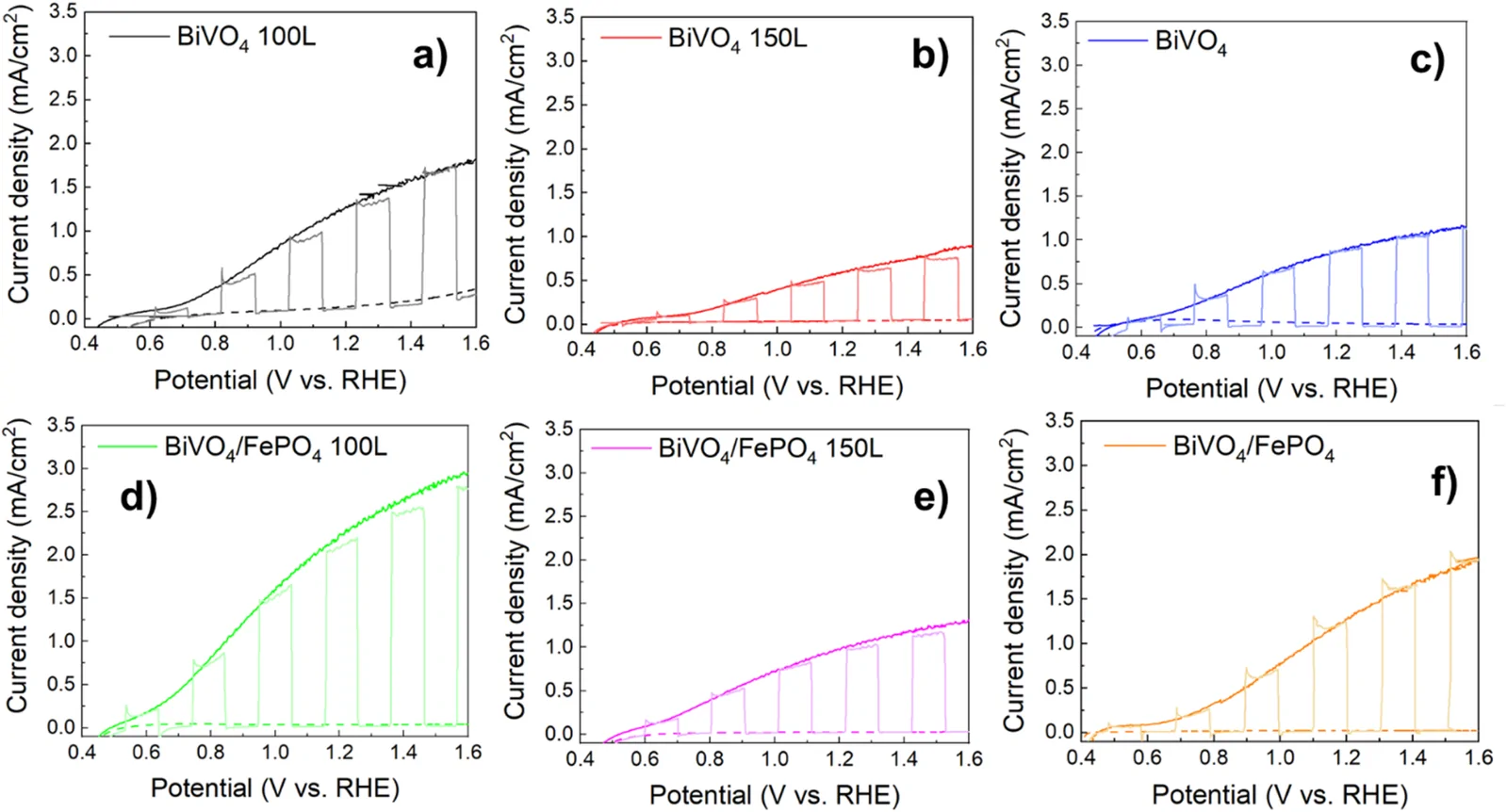
LSV curves showing dark currents (dashed lines), light currents (thick solid lines) and chopped current (solid thin lines): (a) BiVO_4_ 100L; (b) BiVO_4_ 150L; (c) BiVO_4_; (d) BiVO_4_/FePO_4_ 100L; (e) BiVO_4_/FePO_4_ 150L; (f) BiVO_4_/FePO_4_.

The applied bias photon-to-current efficiency (ABPE), calculated from the LSV measurements, is shown in Fig. S5. The BiVO_4_/FePO_4_ 100L photoanode exhibits the highest ABPE values over the investigated potential range, reaching a maximum of approximately 0.43%, confirming the beneficial effect of the MXene modification on the overall PEC performance. Chronoamperometric measurements were conducted at 1.23 V *vs.* RHE under chopped illumination to provide a direct comparison of the photocurrent responses among the different samples (Fig. 7a). All the samples exhibit stable and reproducible photocurrent signals, and the overall trend is consistent with the LSV results, where the potential was linearly increased. The incorporation of FePO_4_ enhances the photocurrent compared to pristine BiVO_4_, and the introduction of a thin MXene overlayer (BiVO_4_ 100L) improves the photoresponse as well. The combined modification with FePO_4_ and an optimally thin MXene layer delivers the best performance, with BiVO_4_/FePO_4_ 100L achieving a steady-state photocurrent exceeding 2 mA cm^−2^, approximately twice that of pristine BiVO_4_ under identical conditions. In contrast, a thicker MXene coating (150 layers) suppresses the photocurrent, as evidenced by the lower performance of both BiVO_4_ 150L compared to BiVO_4_ 100L and BiVO_4_/FePO_4_ 150L compared to BiVO_4_/FePO_4_ 100L. Moreover, the incident photon-to-current conversion efficiency (IPCE) of the BiVO_4_/FePO_4_ 100L photoanode demonstrates a substantial improvement in its overall photoresponse, reaching approximately 16.5% at 450 nm, compared with only ∼8–9% for BiVO_4_/FePO_4_ and unmodified BiVO_4_ at the same wavelength (Fig. S5). This remarkable enhancement is attributed to the synergistic interaction between the FePO_4_ layer and the optimally thin Ti_3_C_2_T_*x*_ MXene overlayer, which promotes more efficient charge separation, facilitates hole extraction, and accelerates interfacial charge transfer during photoelectrochemical water oxidation. Notably, all FePO_4_-containing photoanodes display an anodic peak of the photocurrent immediately upon illumination, followed by a decay of the transient photocurrent to a steady-state value. This transient overshoot is correlated with the accumulation of photogenerated holes at the electrode surface, leading to enhanced surface recombination.^11^ With the introduction of MXene, the accumulation of photogenerated holes at the surface is reduced, indicating that the MXene overlayer facilitates hole transfer and suppresses surface charge recombination. It is important to note that FePO_4_ does not contribute significantly to light absorption and therefore does not directly enhance the IPCE response. Instead, its role is primarily interfacial: amorphous FePO_4_ acts as a hole-accepting layer and redox mediator. In particular, Fe^3+^ species can be oxidized to Fe^4+^ intermediates under operation, which are widely reported as active centers for water oxidation, facilitating O–O bond formation and improving surface reaction kinetics.^58,59^ In parallel, the phosphate matrix contributes to surface passivation and enhanced charge transfer by stabilizing the BiVO_4_ interface and suppressing recombination. This dual function explains the observed increase in photocurrent density despite minimal changes in optical properties.^16^

**Fig. 7:**
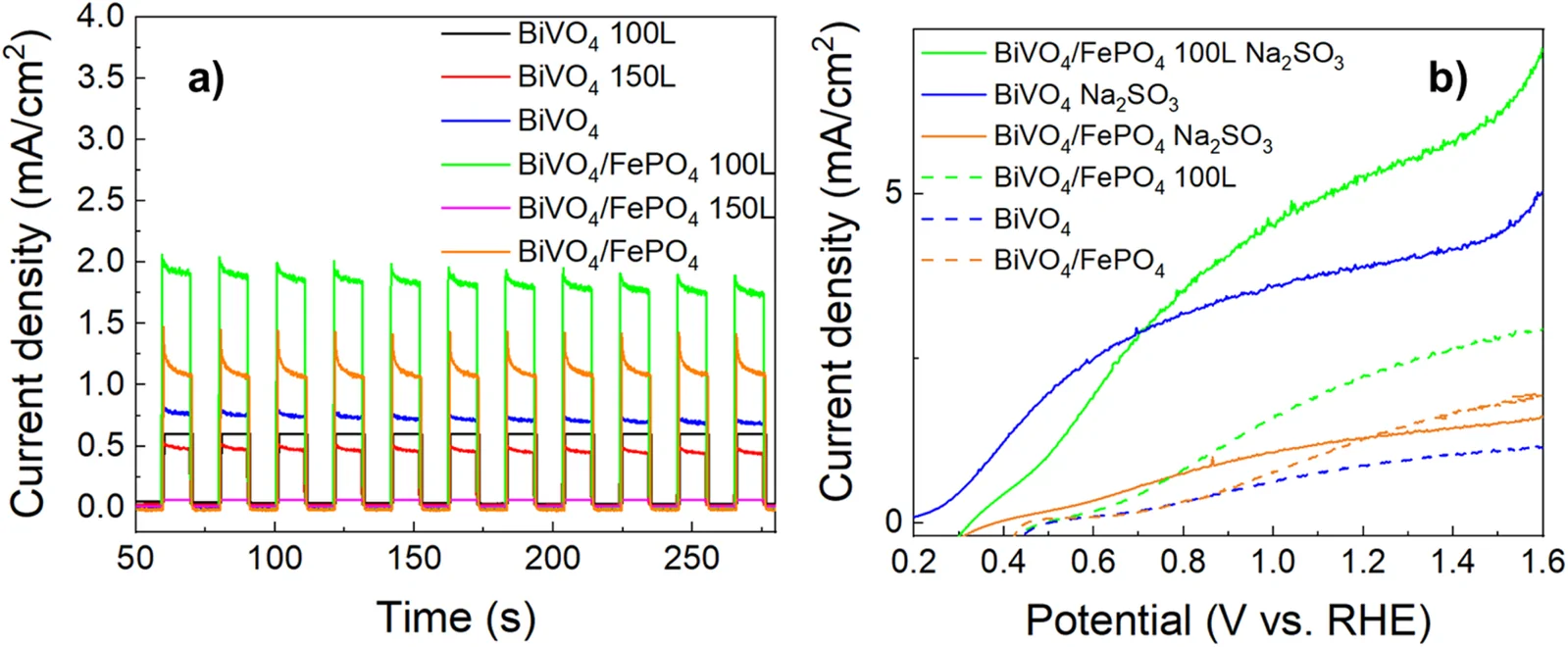
(a) Chronoamperometry of all examined photoanodes at 1.23 V *vs.* RHE; (b) LSV curves showing dark currents (dashed lines) and light currents (thick solid lines) of samples BiVO_4_, BiVO_4_/FePO_4_ and BiVO_4_/FePO_4_ 100L after adding Na_2_SO_3_ as a hole sacrificial agent in the neutral electrolyte.

To further verify the role of surface hole accumulation, a hole sacrificial agent was added in the electrolyte to easily collect the surface holes from the depleted region in the photoanode (Na_2_SO_3_). As shown in Fig. 7b, the addition of sulphite induces a cathodic shift in the photocurrent onset for all samples, indicating the earlier initiation of sulphite oxidation compared to water oxidation. Under these conditions, the photocurrent density of BiVO_4_/FePO_4_ 100L increases significantly to 5.32 mA cm^−2^, approximately 2.3 times higher than that measured in Na_2_SO_4_ alone, confirming that surface recombination substantially limits the water oxidation performance. Furthermore, the LSV curves in both pure Na_2_SO_4_ and Na_2_SO_3_-containing electrolytes reveal that BiVO_4_/FePO_4_ 100L exhibits a noticeably steeper slope than pristine BiVO_4_ and BiVO_4_/FePO_4_, demonstrating accelerated surface oxidation kinetics. Collectively, the transient photocurrent and LSV analyses indicate that the synergistic modification with FePO_4_ and MXene not only enhances surface reaction kinetics but also improves charge carrier density and bulk charge separation, leading to a pronounced enhancement in overall PEC performance. Charge transfer efficiency (*η*_tr_) was calculated at 1.23 V *vs.* RHE to elucidate the specific roles of each component within the heterostructure. Pristine BiVO_4_ exhibits an *η*_tr_ of 23%, indicating moderate interfacial hole transfer efficiency. Upon introducing only the FePO_4_ layer, *η*_tr_ decreases to nearly zero, suggesting that although FePO_4_ effectively passivates surface states and enhances bulk charge separation, it does not sufficiently promote interfacial water oxidation kinetics. As a result, surface hole transfer to water remains limited. In contrast, the incorporation of an optimized MXene overlayer dramatically increases *η*_tr_ to 43%, reflecting significantly improved interfacial hole transfer efficiency. This enhancement indicates that MXene provides an efficient conductive pathway and active interfacial platform for hole extraction, thereby increasing the charge transfer rate constant while suppressing surface recombination. Overall, the combined FePO_4_/MXene modification produces a synergistic effect by coupling improved bulk charge separation (*via* FePO_4_ passivation) with accelerated surface reaction kinetics (*via* MXene-mediated charge transfer), leading to substantially enhanced PEC performance.

#### 3.5.2 EIS & IMPS

For a further investigation of the various charge phenomena of the photoelectrodes, photoelectrochemical impedance spectroscopy (PEIS) was employed at 1.23 V *vs.* RHE (Fig. 8a). As for the influence of MXene, the semicircle of Nyquist plots of the photoanode with thin MXene layers is comparable to that of the reference samples and much smaller than that of the electrodes with thick MXene layers, suggesting that thin MXene effectively reduces the impedance for charge transfer at the electrode–electrolyte interface, while thick MXene increases the resistance thus inhibiting the charge transfer. As for FePO_4_, it contributes to decreasing the impedance as the semicircle of Nyquist plots of EIS of BiVO_4_/FePO_4_ is smaller than that of BiVO_4_. The BiVO_4_/FePO_4_ 100L sample has the lowest charge transfer resistance *R*_ct_, indicating an improvement in interfacial conductivity under the synergetic effect of FePO_4_ and MXene (see details in Table S2). This trend resonates with the PEC properties of the samples. To quantify these interfacial charge transfer properties, the EIS spectra are fitted with the equivalent circuit shown in the inset of Fig. 8a. The *R*_ct_ was reduced from 5917 Ω to 3811 Ω after the modification of BiVO_4_ with FePO_4_ and MXene. Additionally, comparing dark *vs.* light measurements (Fig. S7), it can be confirmed that, upon light exposure, electron–hole pairs are formed and delivered to the photoelectrode/electrolyte interface, where they promote the OER and consequently lower the overall internal resistance, compared to dark conditions. The improved charge-transfer properties are consistent with the enhanced photocurrent observed in the LSV measurements.

**Fig. 8:**
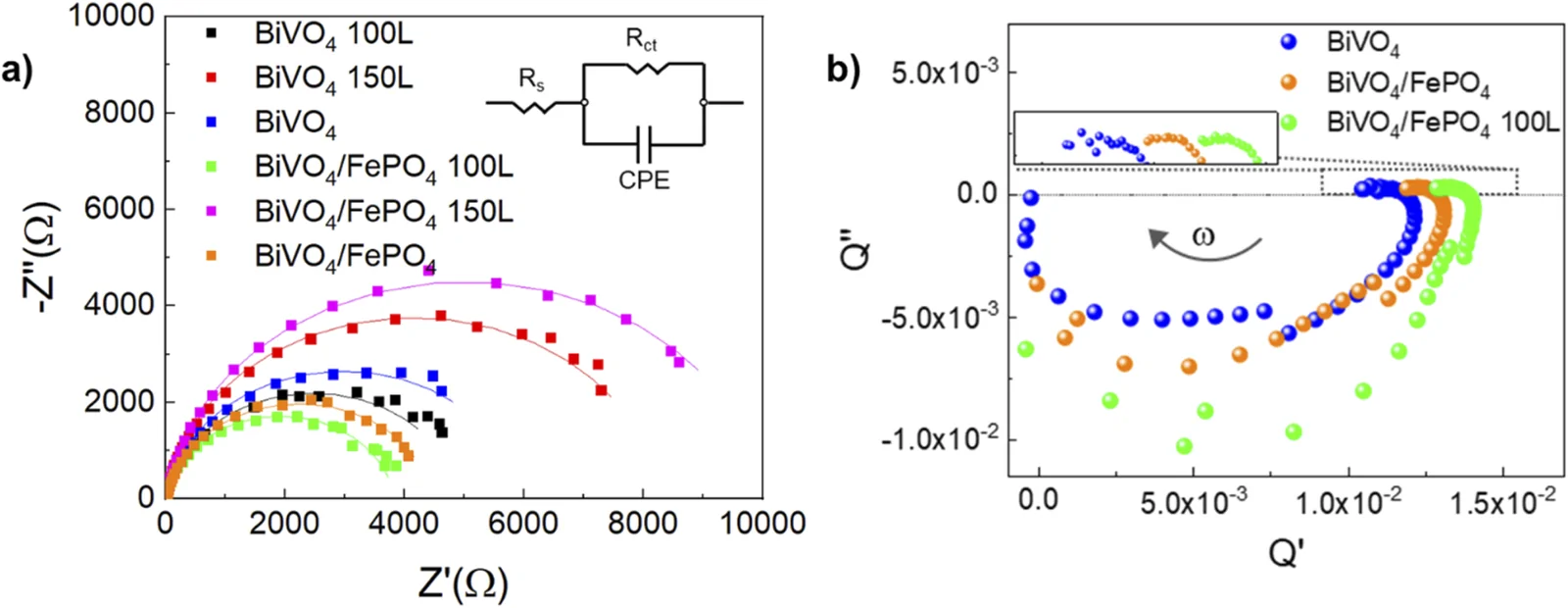
(a) PEIS Nyquist plots at 1.23 V *vs.* RHE. Points are measured data while continuous lines are the Z-fit; (b) IMPS spectra measured in Na_2_SO_4_ 0.5 M at 1.23 V *vs.* RHE. The inset shows the magnification of the low-frequency region located in the first quadrant of the complex plot. Data of samples BiVO_4_/FePO_4_ and BiVO_4_/FePO_4_ 100L are shown down to 1 Hz, while BiVO_4_ data are shown down to 0.2 Hz.

To further elucidate the charge-transfer processes at the photoanode/electrolyte interface and the dynamics of photogenerated carriers, intensity-modulated photocurrent spectroscopy (IMPS) measurements were performed. IMPS probes the photocurrent response to small sinusoidal perturbations in light intensity at a fixed applied potential, with modulation frequencies ranging from 5 kHz to 0.2 Hz. Fig. 8b presents the IMPS spectra of the representative samples recorded at 1.23 V *vs.* RHE. Pristine BiVO_4_, BiVO_4_/FePO_4_, and BiVO_4_/FePO_4_ 100L exhibit well-defined high-frequency semicircles in the fourth quadrant with clear intercepts on the real axis. Notably, the high-frequency intercept of BiVO_4_/FePO_4_ 100L shifts to higher values compared to the other samples, indicating an increased flux of photogenerated holes reaching the semiconductor/electrolyte interface. This observation is consistent with the enhanced PEC performance and the reduced charge-transfer resistance revealed by EIS measurements. At lower frequencies, a semicircle appearing in the first quadrant is typically associated with interfacial processes involving surface charge transfer and recombination.^60–62^ Such features arise when the hole transfer rate is comparable to the recombination rate, reflecting limited charge separation efficiency. The progressive reduction or disappearance of this low-frequency semicircle is commonly observed in systems with optimized interfacial catalysts, at higher applied potentials where charge separation improves, or in the presence of hole scavengers that accelerate hole transfer. In the present study, all three samples display similar low-frequency behavior, with only a slightly more pronounced first-quadrant semicircle for pristine BiVO_4_, suggesting stronger surface recombination and/or slower interfacial hole transfer compared to the FePO_4_- and MXene-modified electrodes. When the frequency range is extended to lower values, BiVO_4_/FePO_4_ and BiVO_4_/FePO_4_ 100L exhibit a progressively flattened response rather than forming a fully closed semicircle (Fig. S8). This behavior may be qualitatively attributed to the distribution of charge-transfer and recombination processes occurring on comparable time scales, without dominance of a single kinetic pathway. Such a distribution likely reflects spatial inhomogeneities in the FePO_4_ and MXene coverage, in agreement with the non-uniform surface composition observed in the SEM–EDX maps (Fig. 2). Overall, the IMPS analysis indicates that the enhanced photocurrent in BiVO_4_/FePO_4_ and BiVO_4_/FePO_4_ 100L photoanodes primarily originates from improved carrier transport and reduced recombination losses prior to hole arrival at the electrolyte interface. The FePO_4_ and MXene layers initially act as surface passivation layers that increase the effective hole flux at the interface. Their deposition moderately enhances interfacial kinetics by facilitating hole transfer; however, further improvement in coating uniformity may help suppress surface recombination more effectively by minimizing the distribution of interfacial surface states.

#### 3.5.3 Stability test

A stability test of BiVO_4_/FePO_4_ 100L was conducted at 1.23 V *vs.* RHE at a light intensity of 100 mW cm^−2^ (Fig. 9a). After approximately 2 hours, the photocurrent retained about 70% of its initial value, indicating stability comparable to previously published work. Then BiVO_4_/FePO_4_ 100L after the stability test was examined to determine whether any noticeable changes had occurred. Morphological analysis revealed no significant variations. The post-reaction SEM image (Fig. 9b) shows that the BiVO_4_/FePO_4_ 100L retains the same characteristics observed prior to PEC testing (Fig. 2c). The MXene flakes remain clearly visible on the surface of BiVO_4_/FePO_4_ 100L. The cross-sectional SEM images revealed no significant morphological differences before and after the PEC measurements (Fig. S9). Furthermore, post-PEC XRD analysis (Fig. S10) showed diffraction peaks identical to those observed prior to the PEC measurements, indicating that the crystalline structure of the material remained unchanged. Then surface-sensitive characterization techniques including Raman spectroscopy and XPS were employed to investigate the surface changes of the photoanodes after PEC operation. The preserved presence and optical signature of MXene flakes on the surface of BiVO_4_/FePO_4_ 100L were further investigated by fiber-optic confocal Raman microscopy. Measurements were carried out under high magnification and at a high numerical aperture (100×, NA = 0.9) using a 785 nm excitation wavelength, deliberately chosen to be off-resonance with the band gap of the dominant BiVO_4_ component while remaining close to an interband transition of Ti_3_C_2_T_*x*_ MXene.^63^ This strictly confocal configuration enhances the optical contribution from MXene flakes while minimizing the BiVO_4_ background. A Raman mapping approach was adopted to account for the superficial inhomogeneity of the MXene coating. As shown in Fig. S11, under white-light illumination, the BiVO_4_/FePO_4_ 100L sample exhibits greater visual heterogeneity than the MXene-free BiVO_4_/FePO_4_ film, displaying patchy darker regions superimposed on the characteristic yellow crystalline BiVO_4_ background. Raman imaging of the region indicated in Fig. 9c reveals that spectra collected from the darker areas (pink spectrum in Fig. 9g) differ markedly from those of the yellow regions (green spectrum in Fig. 9g). Within the spectral window between the VO_4_^3−^ bending doublet (326 and 366 cm^−1^) and the symmetric V–O stretching mode (∼829 cm^−1^), additional broad features appear that are consistent with Ti_3_C_2_T_*x*_ MXene vibrational modes.^64–66^ In particular, the darker areas exhibit (i) a sharp, resonant in-plane skeletal mode at ∼120 cm^−1^, (ii) a broad low-intensity band in the 260–280 cm^−1^ range associated with in-plane vibrations of F- and HO-terminated surface groups, (iii) a broad but more pronounced contribution at around 360–370 cm^−1^ attributed to in-plane modes of surface terminations, (iv) a band at ∼510 cm^−1^ corresponding to the resonant ω_6_ out-of-plane breathing mode of surface groups, (v) a broad feature centered approximately at ∼570, ∼620, and ∼670 cm^−1^ assigned to in-plane carbon lattice vibrations influenced by different degrees of oxidation of the surface groups, and (vi) a relatively sharper band at ∼727–730 cm^−1^ assigned to the resonant out-of-plane ω_3_ mode of carbon atoms, consistent with the flake-like nature and synthesis route of the 2D material.^41,66,67^ Importantly, characteristic BiVO_4_ modes remain visible in these MXene-enriched regions, including the symmetric V–O stretch at ∼829 cm^−1^ and the external lattice mode at ∼210 cm^−1^, with the latter exhibiting a slight asymmetry due to an MXene-related shoulder near 190 cm^−1^ attributed to out-of-plane skeletal vibrations.^41,46,66^ The persistence of BiVO_4_ features confirms the ultrathin nature of the MXene flakes, whose limited thickness prevents complete optical isolation of the layered contributions even under confocal conditions. In contrast, the yellow regions dominated by the BiVO_4_ Raman profile display only a minor broad contribution at ∼640 cm^−1^, attributable to the symmetric stretching of shorter V–O bonds within the vanadate tetrahedra and observed in the pristine BiVO_4_/FePO_4_ sample,^68^ thereby excluding contributions from titanium carbide in those areas. As expected, amorphous FePO_4_ is undetectable under these experimental conditions. For comparison, the MXene-free BiVO_4_/FePO_4_ sample exhibits the typical appearance of BiVO_4_ films, characterized by yellow crystals on a black, yet BiVO_4_-containing, background, as shown in Fig. S12 and S13. All the areas including dark and yellow regions show exclusively the characteristic vibrational modes of BiVO_4_, with no additional spectral features detected.

**Fig. 9:**
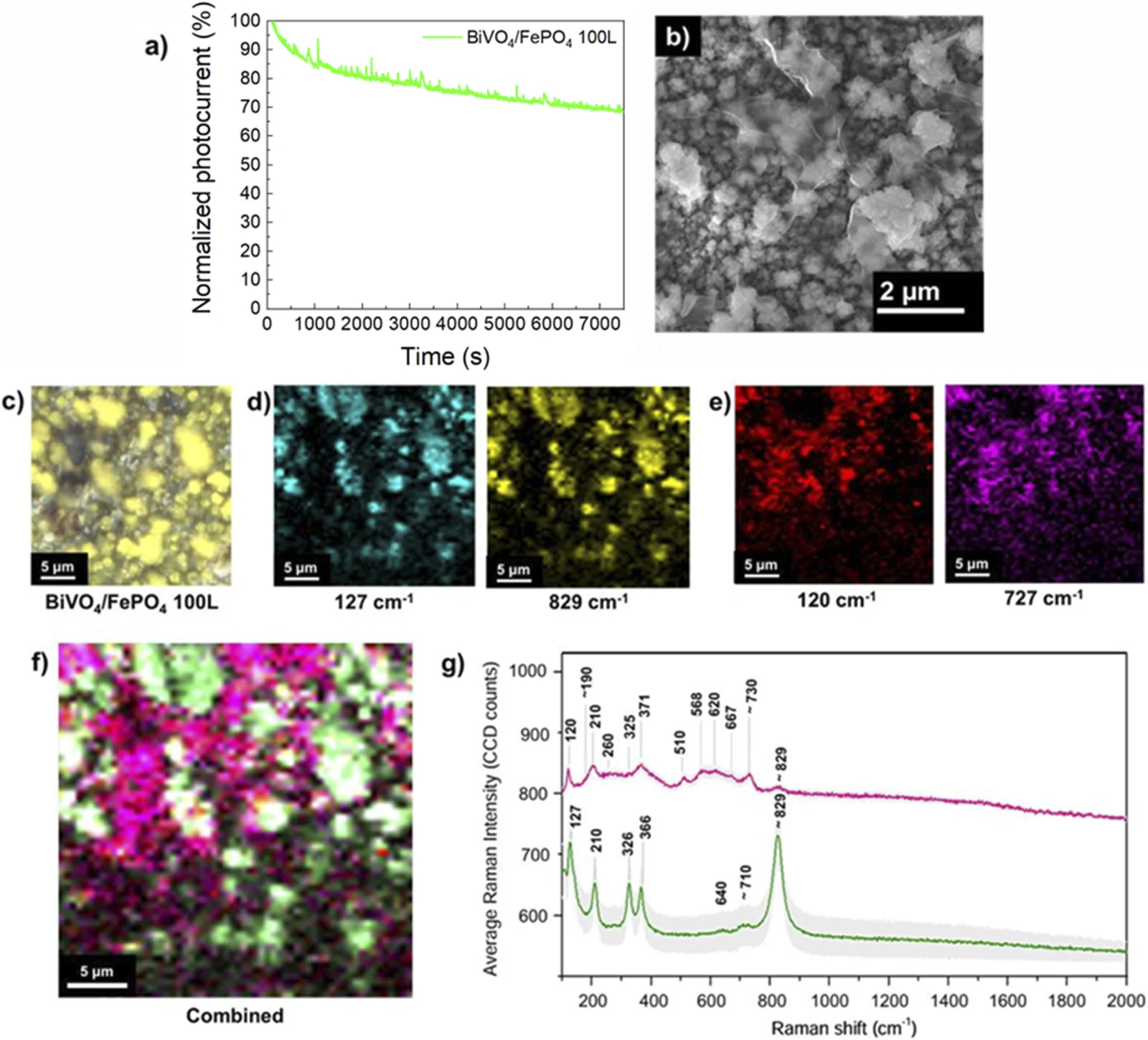
(a) Stability test on BiVO_4_/FePO_4_ 100L performed under illumination at 1.23 V *vs.* RHE; (b) SEM image of BiVO_4_/FePO_4_ 100L post PEC; (c)–(g) Raman imaging (*λ*_exc_ 785 nm) of a representative area of the BiVO_4_/FePO_4_ 100L sample after PEC measurements. (c) White light optical microscopy image of the mapped area. (d) Spatial distribution of the integrated Raman intensities centered at 127 cm^−1^ and 829 cm^−1^ which are utilized as the BiVO_4_ marker bands. (e) Spatial distribution of the integrated Raman intensities centered at 120 cm^−1^ and 727 cm^−1^ which are assigned to Ti_3_C_2_T_*x*_ MXene; (f) combined 127 + 829 + 120 + 733 cm^−1^ channels, highlighting the spatial correlation between the 127 and 829 cm^−1^ bands with the predominantly yellow areas (BiVO_4_), and the 120 + 733 cm^−1^ bands with the darker areas (Ti_3_C_2_T_*x*_ MXene) in the white light optical microscopy image (panel c); (g) average Raman spectra of the two components that emerge from the combined channel map in panel f. The top, pink spectrum is characterized by the MXene signature with residual BiVO_4_ contributions, while the bottom, green spectrum displays BiVO_4_ features only. Shadowed gray bands represent the standard deviation of the mean Raman intensity. Spectra are vertically offset for clarity.

Spatial mapping of the characteristic MXene bands at ∼120 and ∼727 cm^−1^ across the 100L sample is presented in Fig. 9d, while the BiVO_4_-related modes at 127 and 829 cm^−1^ recorded over the same region are shown in Fig. 9e. The MXene-associated signals exhibit higher intensity within the darker areas identified in Fig. 9c, whereas the BiVO_4_ characteristic peaks are more pronounced in the yellow regions. A combination color map incorporating all the bands displayed in Fig. 9d and e is provided in Fig. 8f. This map reveals a coherent spatial distribution that closely follows the darker regions observed under white-light illumination (Fig. 9c) and mirrors the patchy flake morphology observed by SEM (Fig. 2c), thereby confirming the persistent presence of 2D Ti_3_C_2_T_*x*_ MXene on the surface of the BiVO_4_/FePO_4_ 100L sample after PEC operation. Importantly, no additional spectral features indicative of electrochemical oxidation or photothermal degradation are detected. In particular, there is no evidence of TiO_2_ formation (typically appearing at 140–150 cm^−1^) nor of graphitic or amorphous carbon species, which would be characterized by prominent D and G bands at approximately 1390 and 1580–1600 cm^−1^, respectively. These findings, together with the preservation of the characteristic Ti_3_C_2_T_*x*_ MXene vibrational signatures consistent with its original structure, demonstrate that both the BiVO_4_ substrate and the MXene overlayer retain their structural integrity after 2 h of PEC OER operation.

Fig. 10 shows XPS analysis of the BiVO_4_/FePO_4_ 100L photoanode after the PEC stability test. As shown in Fig. 10a, the Ti–C carbide contribution is no longer clearly resolved after PEC testing, while the Ti 2p spectrum becomes dominated by higher-binding-energy Ti species centered at *ca.* 459.0 eV, consistent with TiO_*x*_/TiO_2_-like environments. Consistently, the carbide-related C–Ti component at *ca.* 282.0 eV in the C 1s spectrum disappears after PEC operation, while a weak contribution emerges at *ca.* 288.8 eV, attributable to carbonate species likely originating from electrolyte-related surface residues (Fig. 10b). These results indicate that the outermost MXene-derived surface undergoes substantial oxidation/reconstruction under PEC operating conditions. It is worth mentioning that the apparent discrepancy between XPS and Raman results can be rationalized by considering the different probing depths and sampling volumes of the two techniques. XPS is highly surface-sensitive and probes only the outermost few manometers of the electrode, approximately 6 nm under our experimental conditions. Therefore, the disappearance of the Ti–C/C–Ti components and the dominance of TiO_*x*_/TiO_2_-like signals after PEC testing indicate oxidation/reconstruction of the external surface of the MXene-derived flakes. In contrast, Raman microscopy probes a larger optical volume and remains sensitive to underlying, laterally exposed or partially oxidized MXene-derived domains, which explains the persistence of MXene-related vibrational features in Fig. 9d–f. These observations indicate that PEC operation does not completely remove the MXene-derived overlayer, but induces oxidation of its outermost surface. This surface oxidation is likely one of the causes of the photocurrent decay observed during the 2 h stability test, as the formation of a TiO_*x*_/TiO_2_-like external layer may partially hinder the interfacial charge-transfer function originally provided by the MXene flakes. The Fe 2p and P 2p spectra retain the characteristic features observed before PEC testing, indicating that the FePO_4_ layer is chemically preserved during operation (Fig. 10c and d). Due to the overlap between lattice oxygen, phosphate oxygen, hydroxylated species, adsorbed water and electrolyte-related residues, the O 1s region is used only as complementary evidence (Fig. 10e). Moreover, the quantitative XPS analysis (Table S3) indicates a decrease in the relative surface Ti contribution together with corresponding increases in the relative Bi, V, Fe and P contributions after the PEC stability test. Although these values should not be interpreted as rigorous stoichiometric quantities, they are consistent with partial oxidation and surface reconstruction of the MXene-derived overlayer, resulting in greater XPS sensitivity to the underlying BiVO_4_/FePO_4_ layers. This surface reconstruction is likely to contribute to the gradual photocurrent decay observed during the 2 h stability test.

**Fig. 10:**
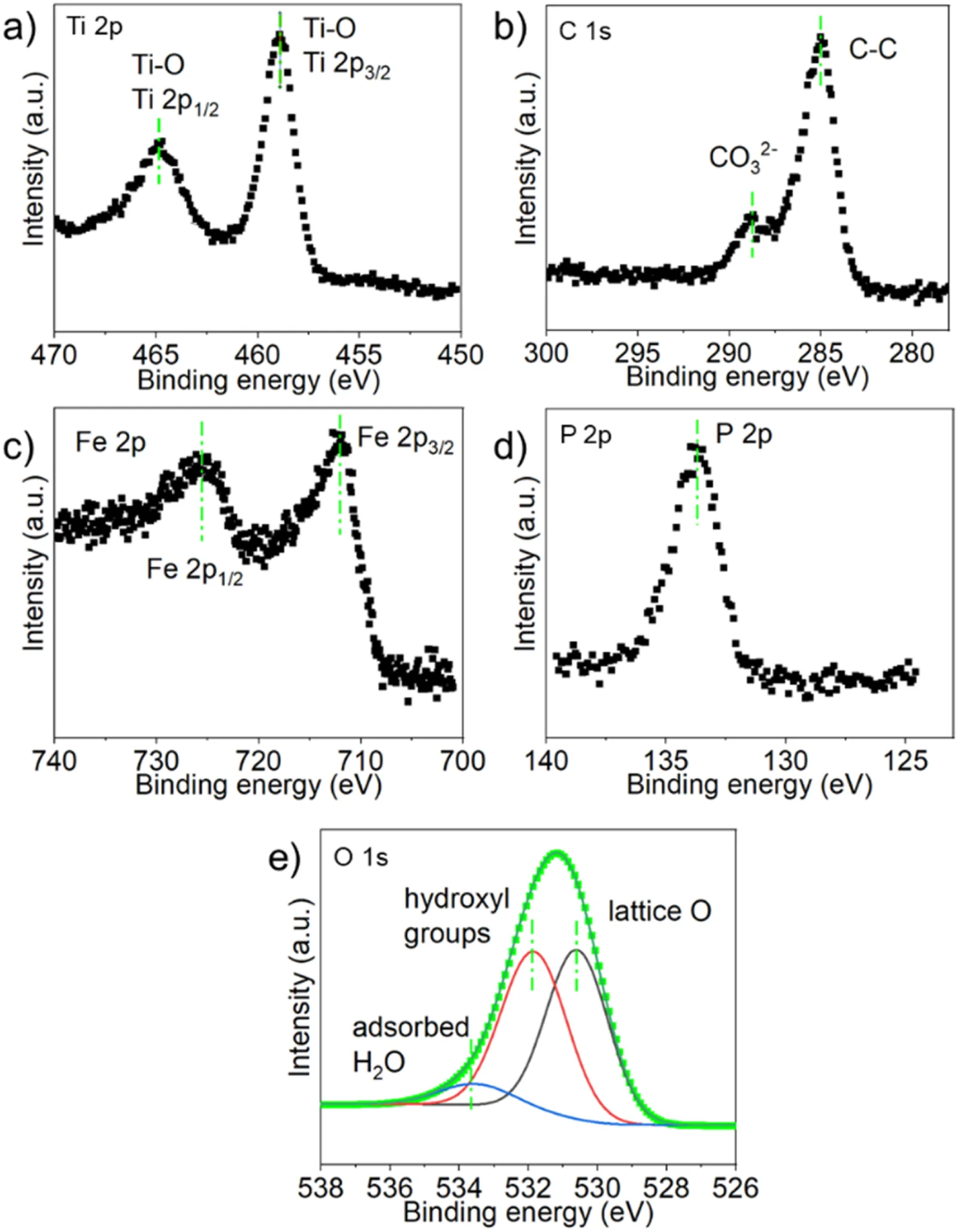
High resolution XPS spectra of BiVO_4_/FePO_4_ 100L after the stability test: (a) Ti 2p, (b) C 1s, (c) Fe 2p, (d) P 2p and (e) O 1s.

### 3.6 Mechanism study

Based on the above results and analysis, the proposed PEC water oxidation mechanism for the BiVO_4_/FePO_4_/MXene photoanode is illustrated in Fig. 11. The system consists of a surface composite/overlayer configuration, where crystalline BiVO_4_ is sequentially coated with an amorphous FePO_4_ overlayer and a Ti_3_C_2_T_*x*_ MXene surface layer, forming well-defined interfaces for charge transfer. As an n-type semiconductor, BiVO_4_ possesses a flat band potential located close to its conduction band (Table 1). Upon modification with FePO_4_ and MXene, the *E*_fb_ shifts progressively toward more positive values, from 0.12 V *vs.* RHE for pristine BiVO_4_ to 0.19 V *vs.* RHE for BiVO_4_/FePO_4_ and further to 0.24 V *vs.* RHE for BiVO_4_/FePO_4_ 100L, while it shifts back to more negative values (0.14 V *vs.* RHE) for BiVO_4_/FePO_4_ 150L (Table 1 and Fig. 11). This anodic shift reflects a downward movement of modified interfacial energetics, resulting in adjusted band alignment and enhanced band bending at the semiconductor/electrolyte interface. Under illumination, photogenerated electrons in the conduction band of BiVO_4_ are driven by the internal electric field toward the back contact and subsequently participate in the hydrogen evolution reaction at the cathode. Simultaneously, *E*_fb_ equilibration between the BiVO_4_/FePO_4_/MXene photoanode and the electrolyte establishes interfacial band bending that promotes directional electron transport toward the cathode and facilitates charge separation. Meanwhile, the valence band edge shifts slightly toward more negative potentials (from 2.74 V *vs.* RHE for BiVO_4_/FePO_4_ to 2.64 V *vs.* RHE for BiVO_4_/FePO_4_ 100L), consistent with the small reduction in the optical band gap after surface modification. The photogenerated holes in the valence band of BiVO_4_ are efficiently transferred across the interfaces first to the FePO_4_ layer and subsequently to the Ti_3_C_2_T_*x*_ MXene overlayer, where water oxidation occurs. Together with the applied electric field, this stepwise hole extraction pathway suppresses surface recombination and promotes efficient hole utilization for the OER. Within this heterostructure, FePO_4_ and Ti_3_C_2_T_*x*_ MXene act as effective hole-accepting and transport layers. MXene enhances interfacial conductivity, accelerates charge transfer, and provides additional active sites for the OER, while FePO_4_ functions both as a hole reservoir for passivation and as a cocatalyst, with Fe^3+^ species serving as catalytically active centers.^16,17^ In addition, MXene slightly improves light harvesting and broadens the photoresponse range, as evidenced by the LSV results (Fig. 6) and UV-vis spectra (Fig. 4). Moreover, an optimally thin MXene overlayer maintains adequate exposure of the underlying FePO_4_ surface, enabling balanced interfacial charge transfer between FePO_4_ and MXene and thereby optimizing their synergistic contribution to the PEC OER. In contrast, excessive MXene loading reduces light absorption, partially shields the FePO_4_ and BiVO_4_ layers, weakens interfacial coupling, and ultimately diminishes the overall PEC performance.

**Fig. 11:**
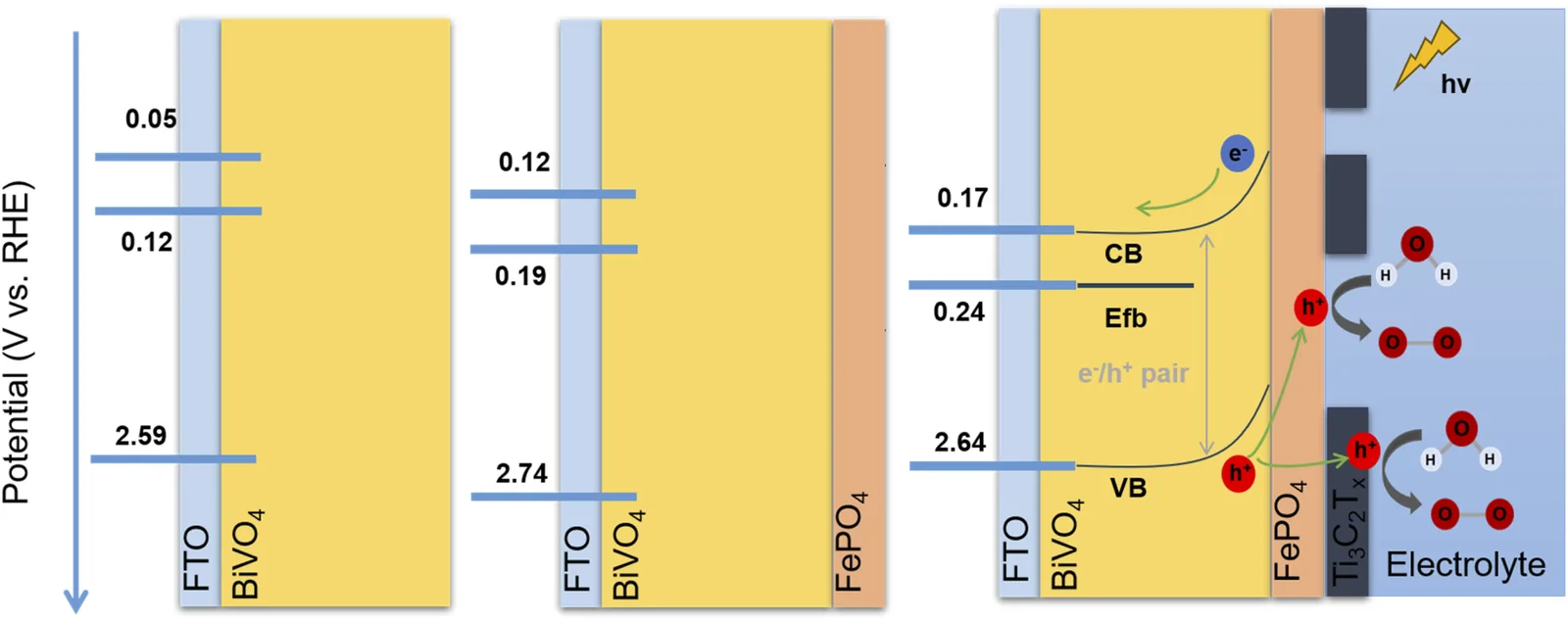
Scheme for the proposed PEC water oxidation mechanism for the optimized BiVO_4_/FePO_4_/MXene photoanode.

## 4 Conclusions

In this work, heterostructured photoanodes for the OER were fabricated based on BiVO_4_ and BiVO_4_/FePO_4_ prepared *via* the Autodrop method, followed by the deposition of Ti_3_C_2_T_*x*_ MXene layers with controlled thickness using an automated spray coating technique. Comprehensive PEC characterization demonstrates that the synergistic integration of FePO_4_ and MXene significantly enhances water oxidation performance. Among the investigated samples, the BiVO_4_/FePO_4_ 100L electrode exhibits the highest photocurrent density. The photoanode retains approximately 70% of its initial photocurrent after 2 h of PEC OER operation, demonstrating moderate operational stability. However, XPS analysis reveals partial oxidation of the MXene overlayer, indicating that additional strategies aimed at stabilizing the MXene surface or controlling its oxidation could further enhance the long-term durability of the photoanode. Mechanistic analysis reveals that both FePO_4_ and MXene promote efficient charge separation: photogenerated electrons are effectively driven toward the cathode, while holes are extracted and transferred to the surface to participate in the OER. An optimized, ultrathin MXene overlayer ensures sufficient exposure of the underlying FePO_4_, where Fe^3+^ species act as active co-catalytic sites for the OER. The enhanced performance is attributed to the combined effect of the FePO_4_ interlayer and the MXene overlayer, which provide complementary functions in the photoanode architecture. While the FePO_4_ layer improves the interfacial properties of BiVO_4_ and facilitates hole transport, the conductive MXene nanosheets promote more efficient interfacial hole transfer between the electrode/electrolyte. In contrast, excessive MXene loading partially shields the active interface and limits performance.

Overall, these findings establish using FePO_4_ and MXene overlayers as an effective strategy to boost the performance of BiVO_4_-based photoanodes and suggest that future efforts should focus on fine-tuning MXene thickness and composition, as well as extending this automated approach to scalable production for solar fuel devices. The enhanced performance can be attributed to the complementary roles of the FePO_4_ interlayer and the MXene overlayer, which together facilitate efficient charge separation and interfacial charge transfer. The use of Autodrop and automated spray deposition provides a practical route toward reproducible fabrication of MXene-modified photoelectrodes. These findings highlight the potential of combining conductive MXene nanosheets with catalytic interlayers as a versatile strategy for improving interfacial charge transport in semiconductor photoanodes and advancing scalable solar fuel technologies.

## Conflicts of interest

There are no conflicts to declare.

## Supplementary Material

NA-008-D6NA00469E-s001

## Data Availability

Additional datasets supporting this work have been included as part of the supplementary information (SI). The datasets generated and analyzed during this work can be made available from the authors on request. Supplementary information (SI) is available. See DOI: https://doi.org/10.1039/d6na00469e.
