# Supplementary material for: MXene-modified BiVO4/FePO4 photoanodes for solar oxygen evolution
Source: Nanoscale Adv. 2026 Aug 18;8(20):5890–907. doi: 10.1039/d6na00469e (PMC13548082; doi:10.1039/d6na00469e)
Supplement: Supplementary file 1 [file NA-008-D6NA00469E-s001.pdf]

Supplementary Information (SI) for Nanoscale Advances.
This journal is © The Royal Society of Chemistry 2026

Supporting Information for:

MXene-modified $BiVO_4/FePO_4$ photoanodes for solar oxygen evolution

Micaela Pozzati,[a] Chiara Deriu,[a] Paola Ragonese,[b] Francesco Lamberti,[c] Mattia Cattelan,[c] Veronika Zahorodna,[d,e] Roberto Altieri,[f] Yana Ihnatenko[e], Serhii Dukhnovskiy,[d, e] Ivan Baginskiy,[d, e] Oleksiy Gogotsi,[d, e] Isabella Poli,[b] Laura Fabris,[a] Teresa Gatti [a,f,*] and Mengjiao Wang[a,*]

[a] Department of Applied Science and Technology, Politecnico di Torino, C.so Duca degli Abruzzi 24, 10129 Torino, Italy

[b] Center for Sustainable Future Technologies, Istituto Italiano di Tecnologia, Via Livorno 60, 10144 Torino, Italy

c Dipartimento di Scienze Chimiche, Università degli Studi di Padova, Via Marzolo, 1, 35131, Padova, Italy

[d] Materials Research Centre, 3 Krzhizhanovskogo Str., 03142 Kyiv, Ukraine

[e] Y-Carbon Ltd., 18 Bohdana Havrylyshyna st, Kyiv, Ukraine

[f] Center for Materials Research, Justus-Liebig University Giessen, Heinrich-Buff-Ring 16-17, 35392 Giessen, Germany

E-mail: teresa.gatti@polito.it ; mengjiao.wang@polito.it

Table of Contents

- Automated spray coating system used for MXene coated samples preparation (Figure S1).
- XRD patterns of $BiVO_4/FePO_4$, $BiVO_4/FePO_4$ 100L, and $BiVO_4/FePO_4$ 150L samples and reference pattern of $BiVO_4$ monoclonic scheelite phase of $BiVO_4$ (Figure S2).
- Single-point Raman spectra of all $BiVO_4/FePO_4$/MXene samples (Figure S3).
- Survey scanning obtained on $BiVO_4/FePO_4$ 100L (Figure S4).
- Comparison with $BiVO_4$-based photoanodes regarding photocurrent density, ABPE, stability and fabrication method (Table S1).
- ABPE for $BiVO_4$, $BiVO_4/FePO_4$ and $BiVO_4/FePO_4$ 100L samples (Figure S5).
- IPCE of all photoanode samples at 1.23V vs. RHE (Figure S6).
- PEIS Nyquist plot in dark vs light of $BiVO_4/FePO_4$ 100L sample at 1.23V vs. RHE (Figure S7).
- Charge transfer resistance values obtained at 1.23V vs. RHE (Table S2).
- First quadrant of the complex plot of the IMPS spectra measured in 0.5 M Na_2SO_4 at 1.23 V vs RHE. Data are shown down to 0.2 Hz. (Figure S8).
- XRD pre and post PEC of $BiVO_4/FePO_4$ 100L sample (Figure S9).

- Cross-section SEM of $BiVO_4/FePO_4$ 100L a) pre PEC b) post PEC (Figure S10).
- White light optical micrographs (100x) of $BiVO_4/FePO_4$ and $BiVO_4/FePO_4$ 100L (Figure S11).
- Raman imaging (λ_{exc} 785 nm) of a representative area of the $BiVO_4/FePO_4$ sample after PEC measurements (Figure S12).
- Raman spectra (λ_{exc} 785 nm) extracted from 7 points across a representative area of a pristine $BiVO_4$ sample (Figure S13).

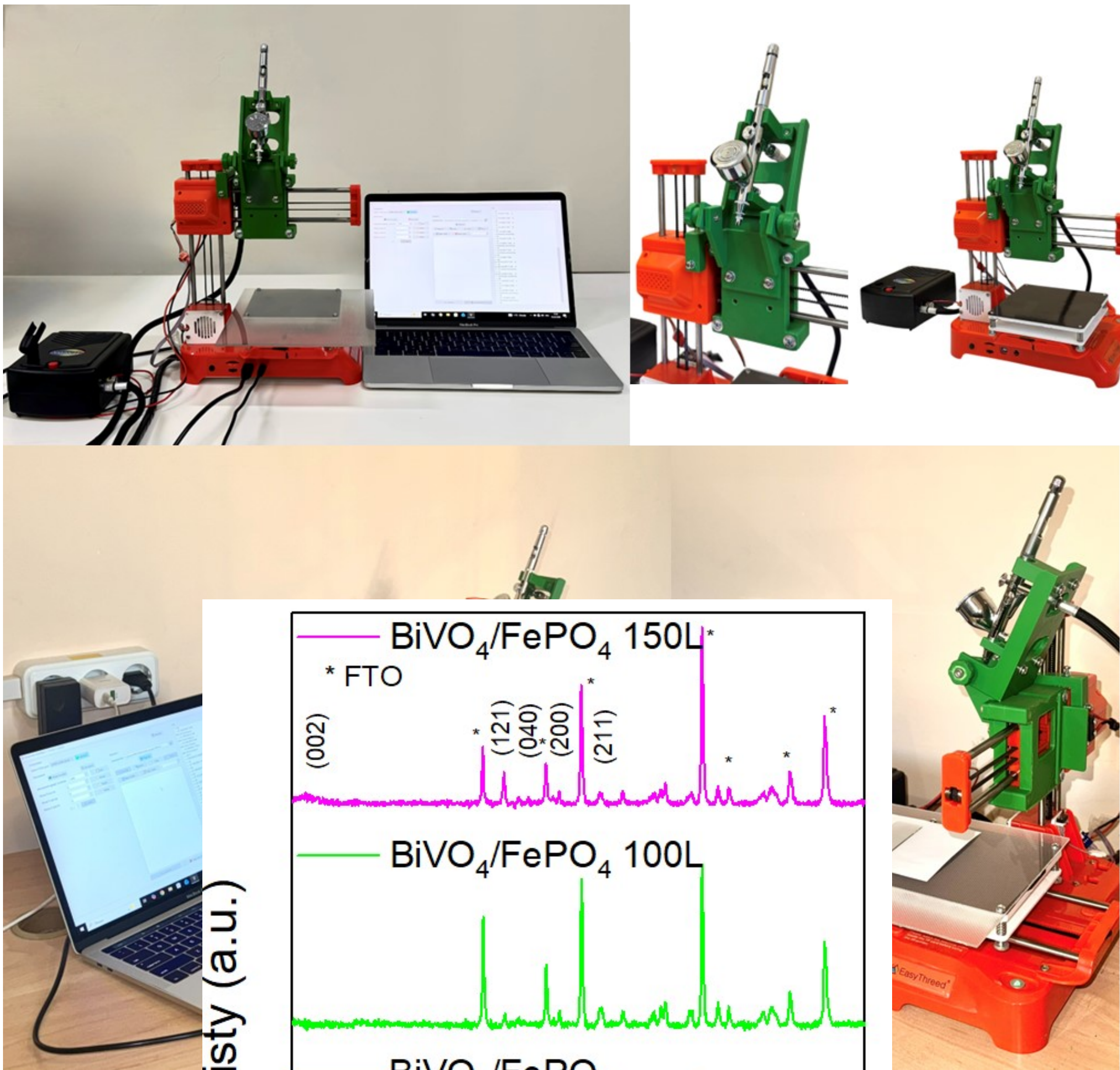

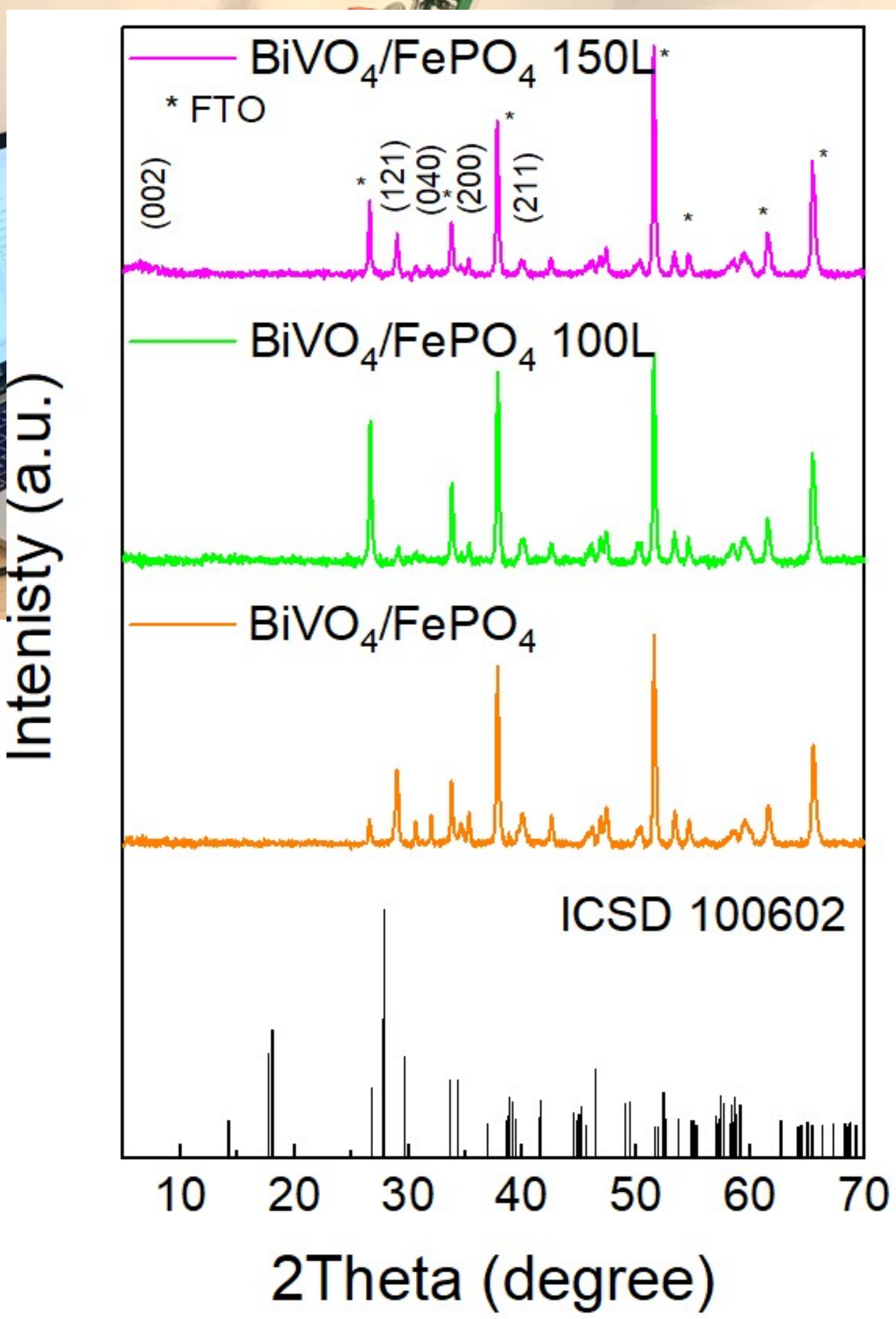

Fig. S1. Automated spray coating system used for MXene-coated samples preparation.

Fig. S2. XRD patterns of $BiVO_4/FePO_4$, $BiVO_4/FePO_4$ 100L, and $BiVO_4/FePO_4$ 150L samples and reference pattern.

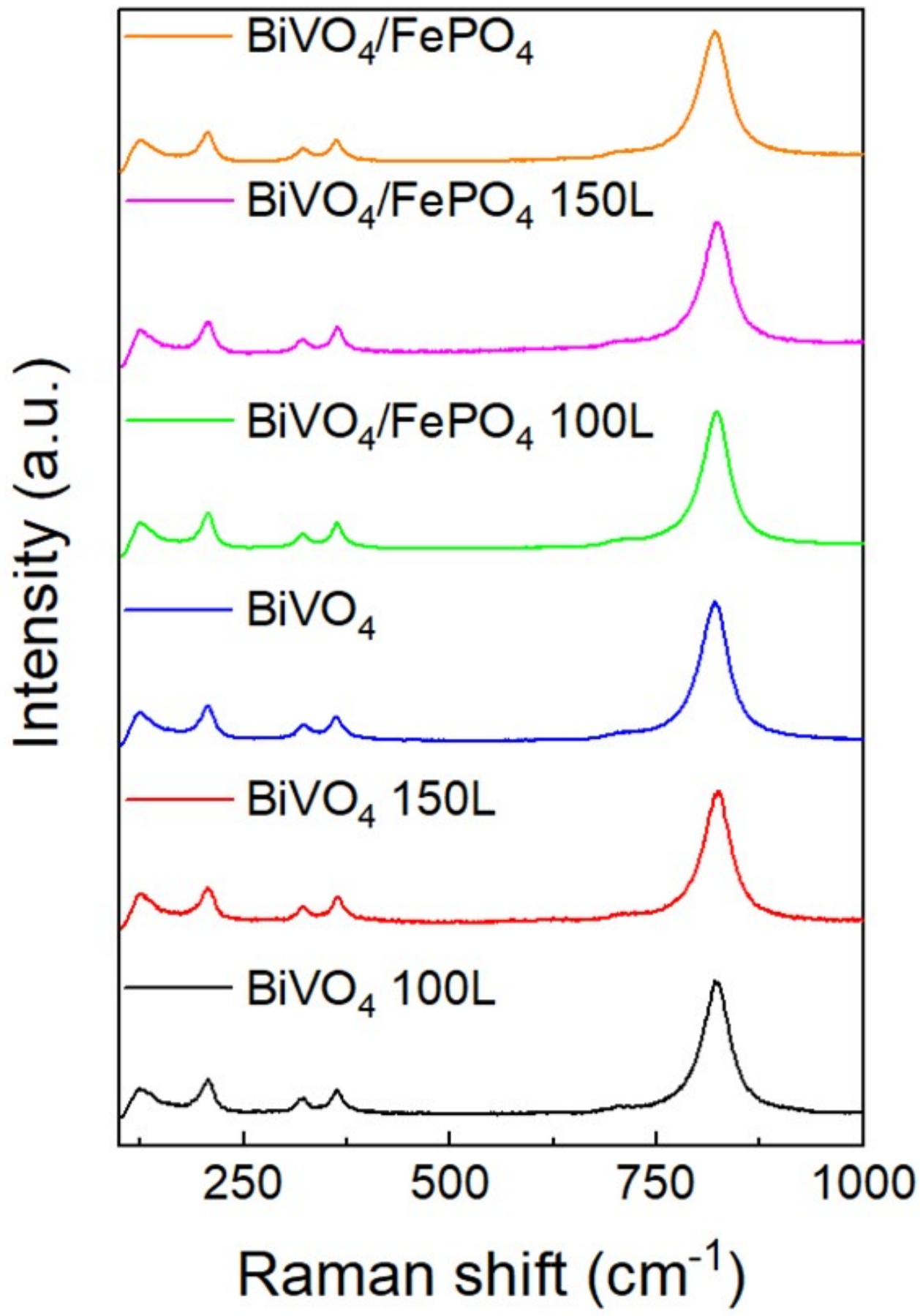

Fig. S3. Single-point Raman spectra of all $BiVO_4/FePO_4$/MXene samples.

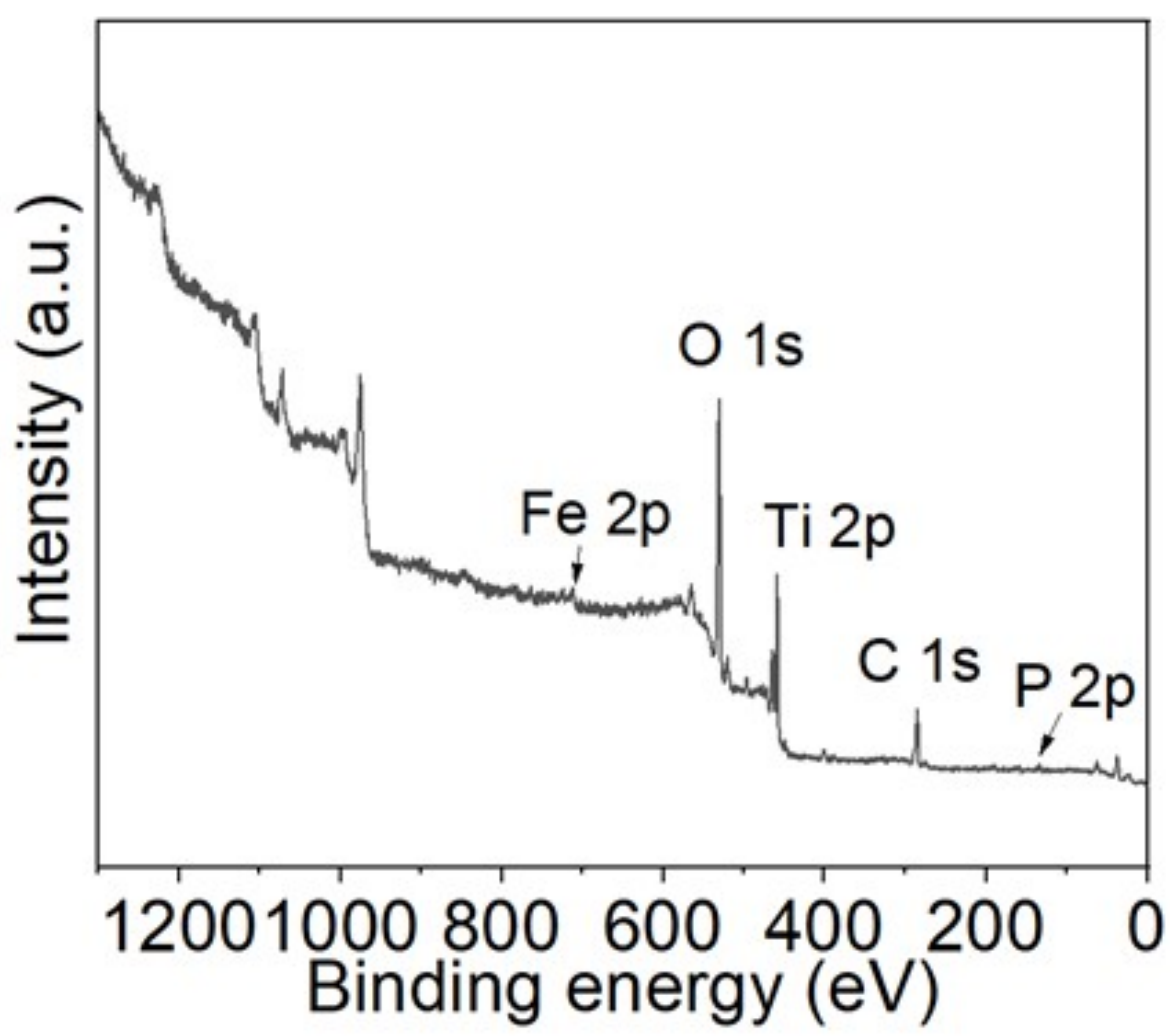

Fig. S4. Survey scanning obtained on $BiVO_4/FePO_4$ 100L.

Table S1. Comparison with $BiVO_4$-based photoanodes regarding photocurrent density, ABPE, stability and fabrication method.

System	J @ 1.23 V (mA/cm²)	ABPE (%)	Stability	Fabrication method
**$BiVO_4$/Ferrihydrite/MXene**	~4.55	~1.57	92.5% after 3h	Hydrothermal reaction[55]
**$BiVO_4$/Co,Fe-NTMP**	~2.6	~0.58	Stable photocurrent after 2.5h	Electrodeposition + immersion[4]
**Co-Pi/V_2CT_x/$BiVO_4$**	~4	~1.55	Initial photocurrent up to 10h	Electrodeposition[17]
**B/$BiVO_4$/NiFeOx**	~2.45	~0.77	44% after 5h	s-SILAR method[57]
**$BiVO_4$/FeOOH/CACo**	~3	~0.70	Stable photocurrent after 10h	Electrodeposition + immersion[56]
**$BiVO_4$/$FePO_4$/MXene (this work)**	~2.3	~0.43	70% after 2h	Autodrop + Mxene spray coating

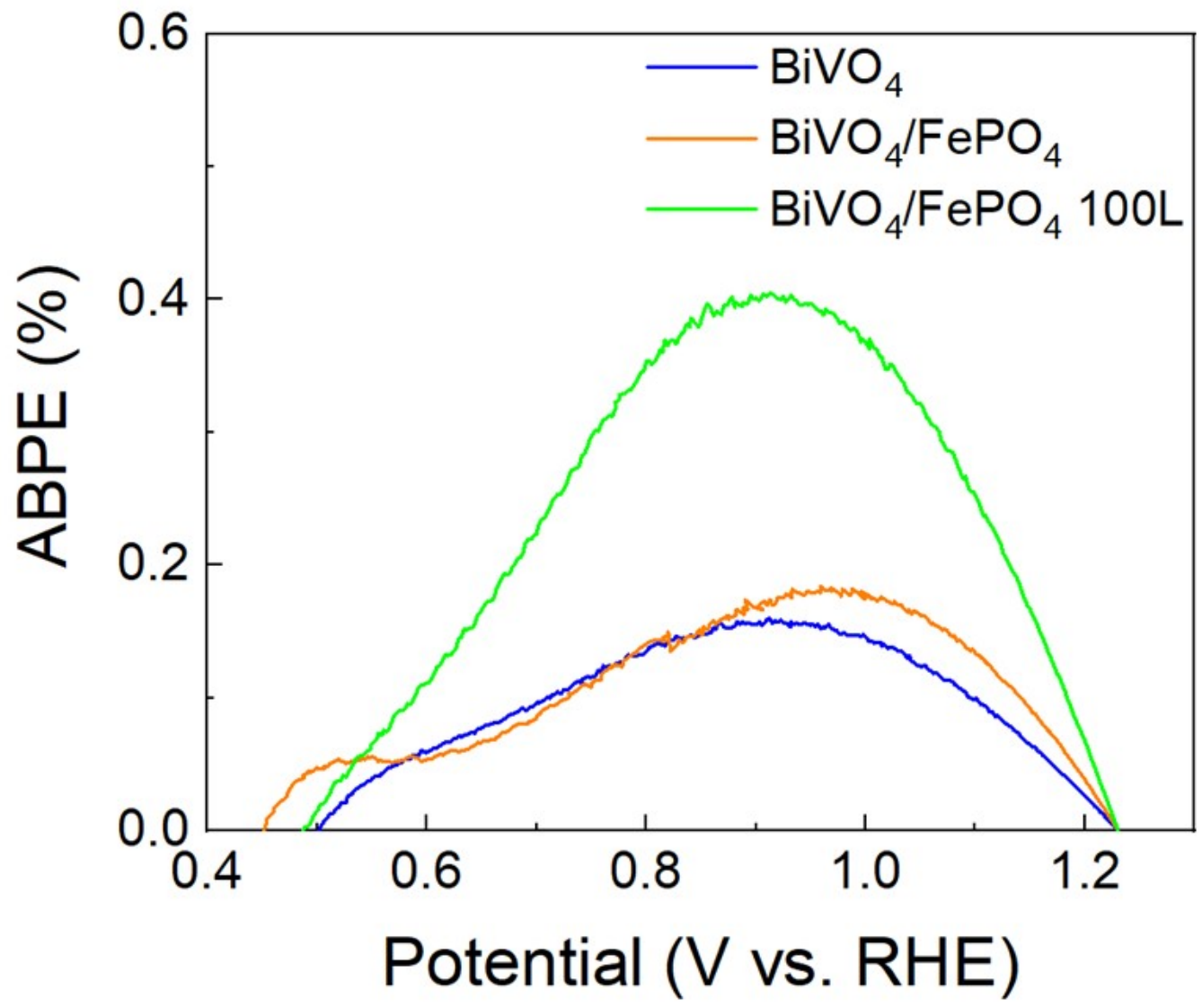

Fig. S5. ABPE for $BiVO_4$, $BiVO_4$/$FePO_4$ and $BiVO_4$/$FePO_4$ 100L samples.

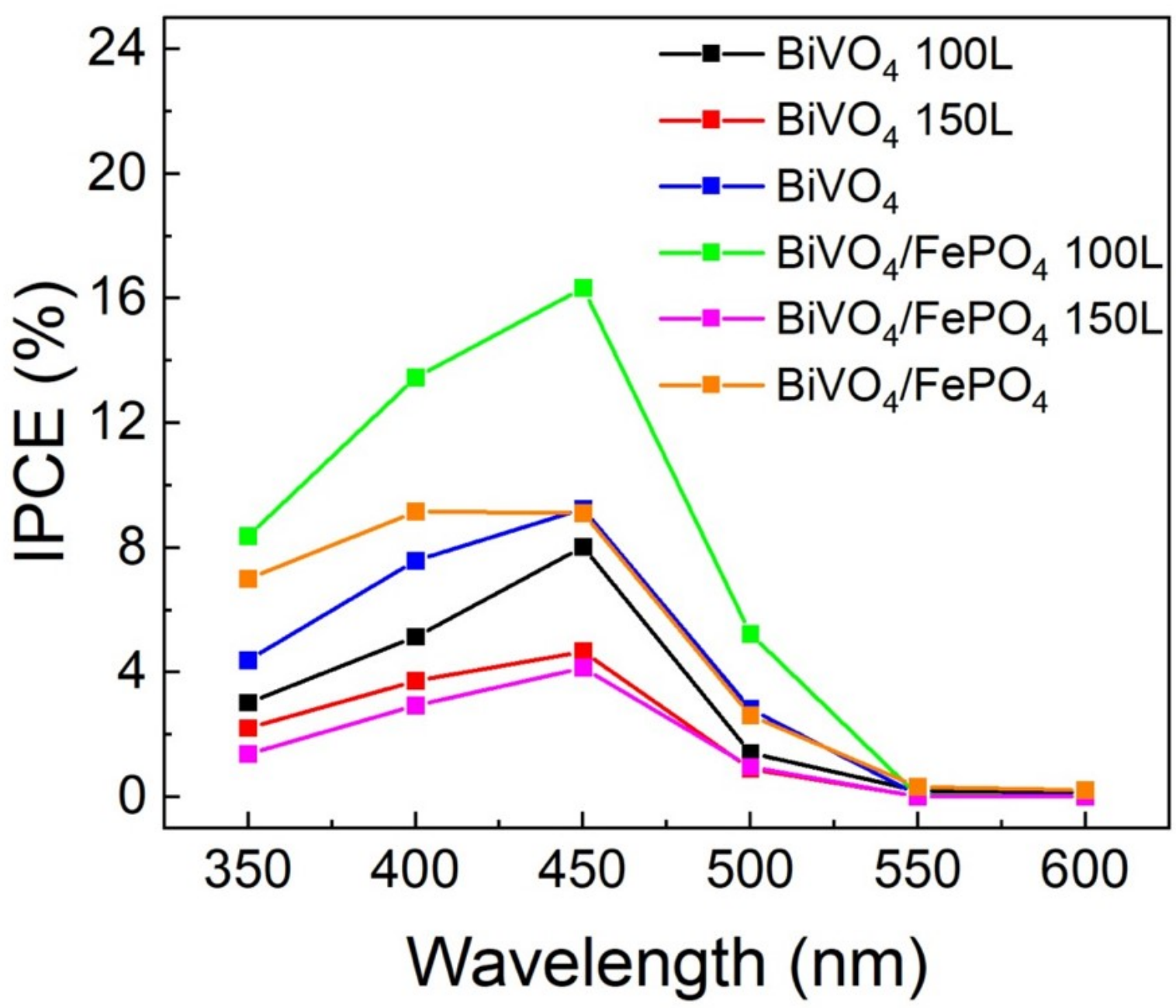

Fig. S6. IPCE at 1.23V vs. RHE for all photoanode samples.

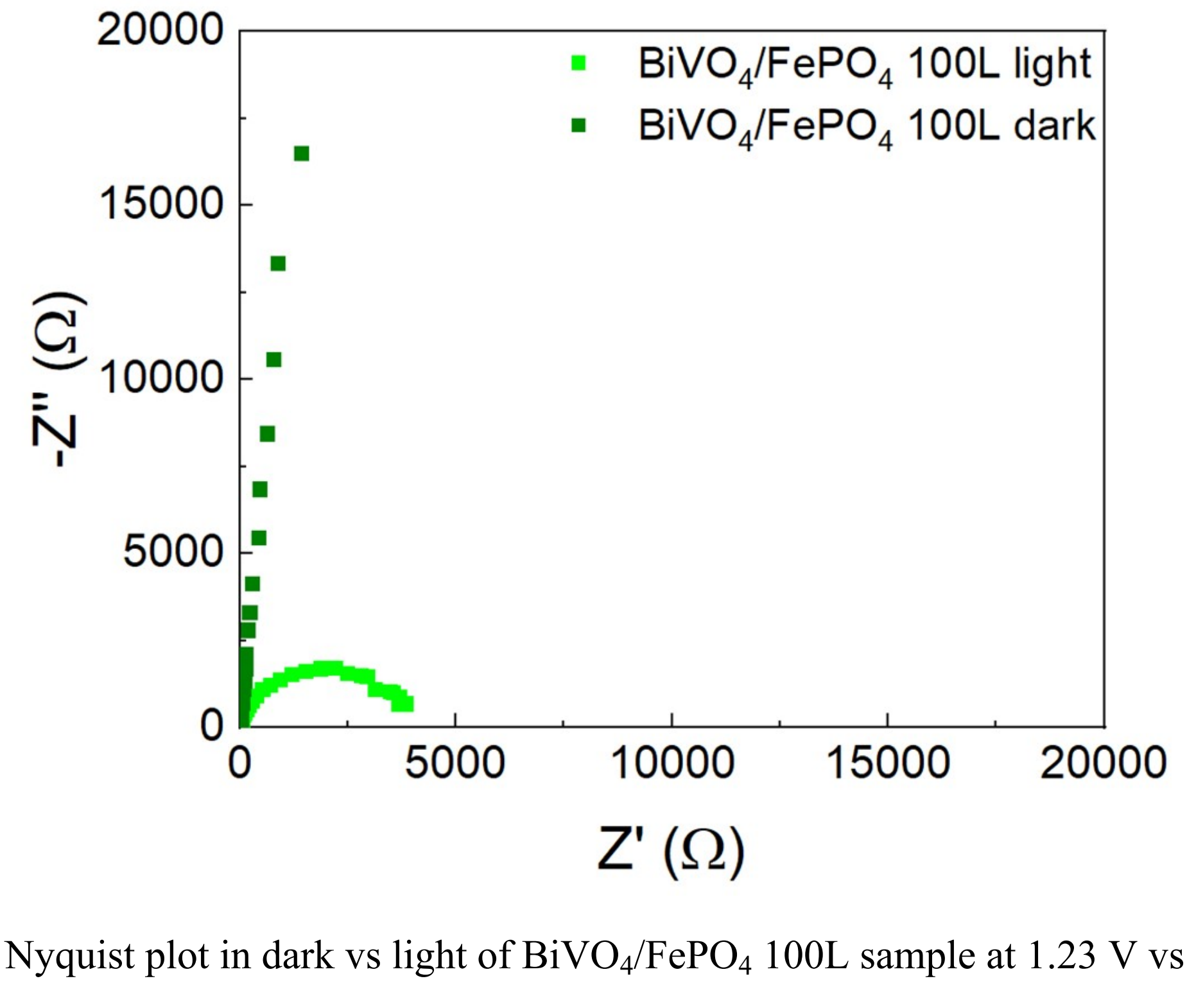

Fig. S7. PEIS Nyquist plot in dark vs light of $BiVO_4/FePO_4$ 100L sample at 1.23 V vs. RHE.

Table S2. Charge transfer resistance values obtained at 1.23 V vs. RHE.

Sample	$BiVO_4$ 100L	$BiVO_4$ 150L	$BiVO_4$	$BiVO_4/FePO_4$ 150L	$BiVO_4/FePO_4$ 100L	$BiVO_4/FePO_4$
**R_{ct} (Ω)**	4902	8266	5917	10023	3811	4445

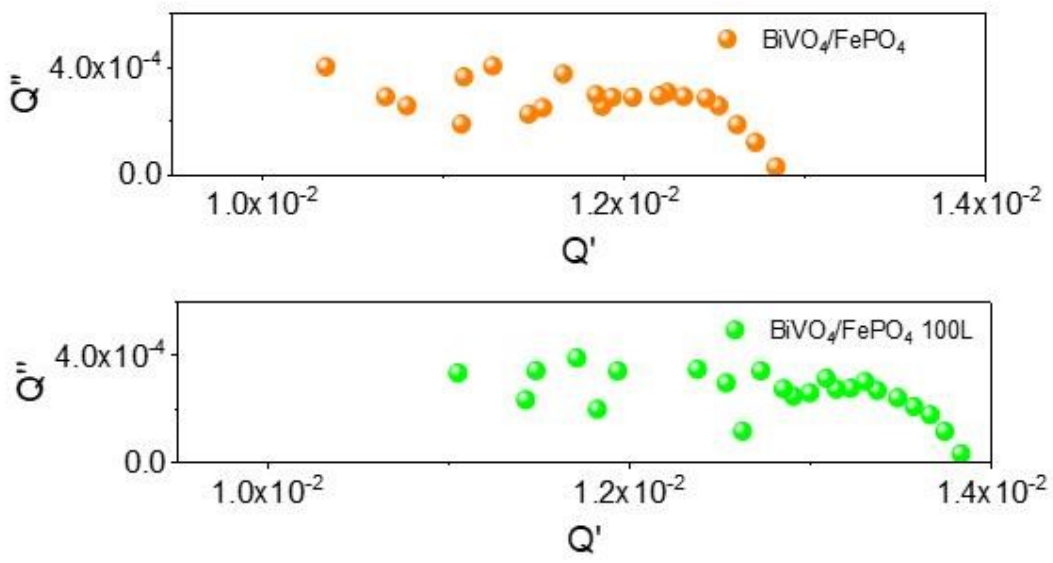

Fig. S8. First quadrant of the complex plot of the IMPS spectra measured in 0.5 M Na_2SO_4 at 1.23 V vs RHE. Data are shown down to 0.2 Hz.

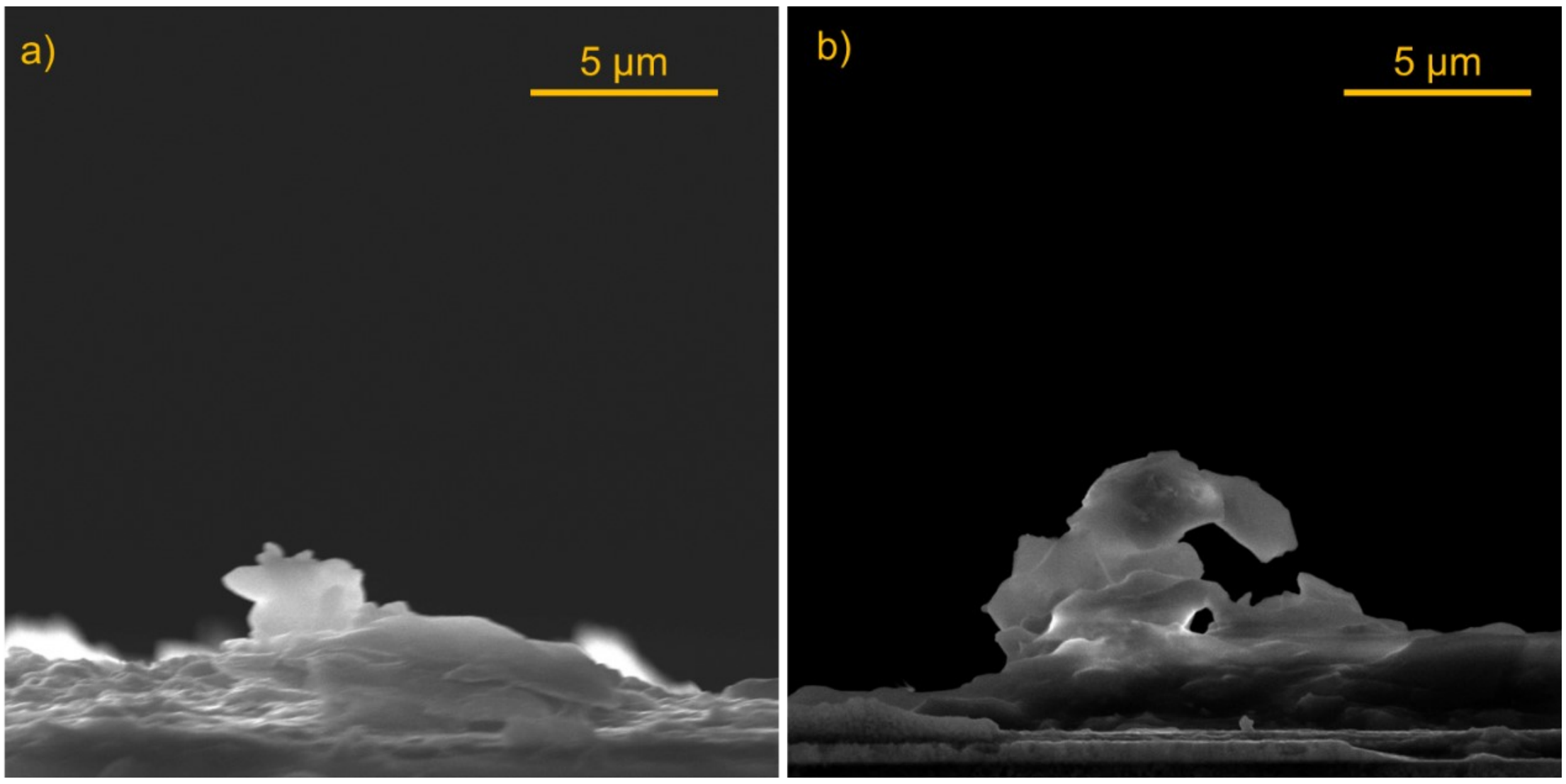

Fig. S9. Cross-section SEM of BiVO4/FePO4 100L a) pre PEC b) post PEC.

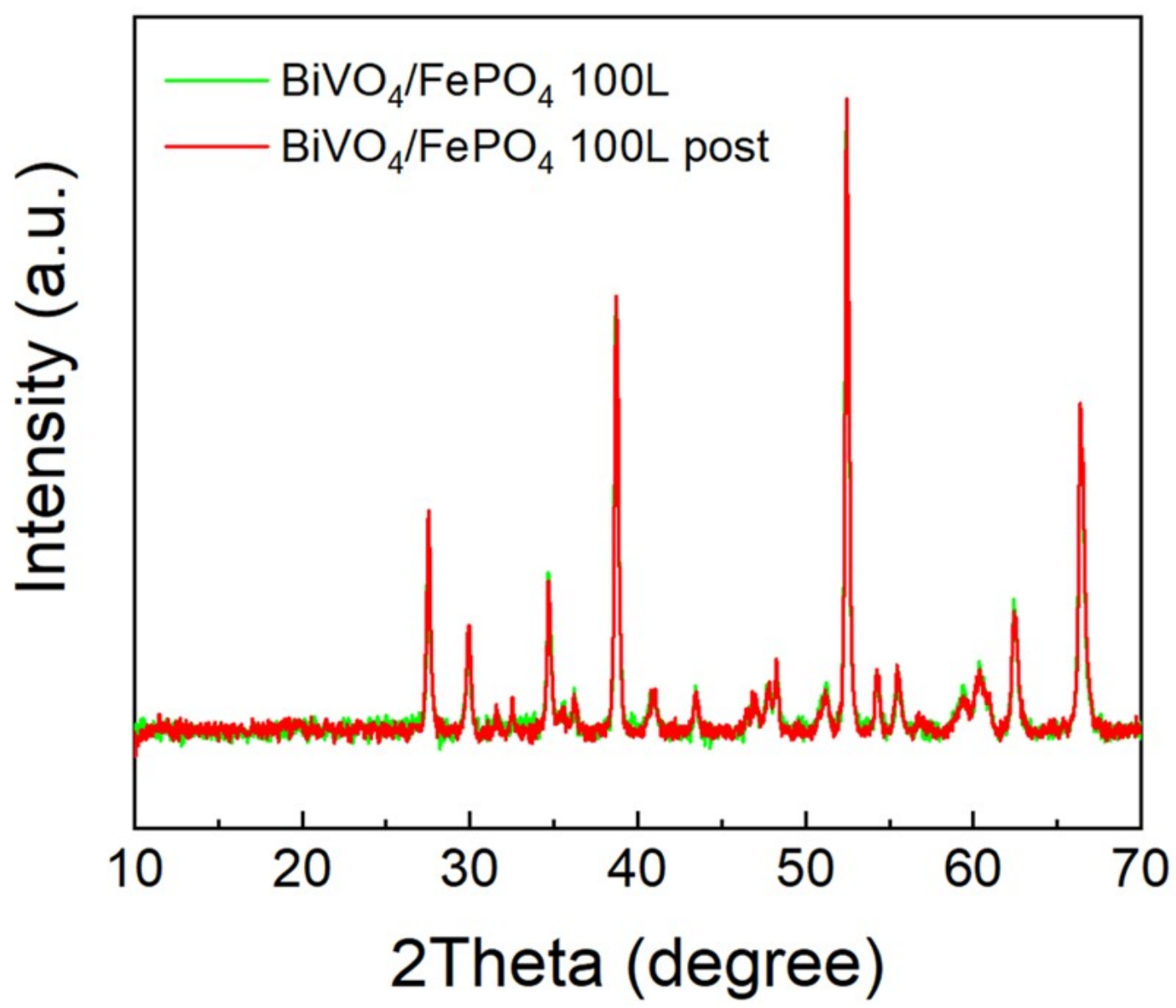

Fig. S10. XRD pre and post PEC of $BiVO_4/FePO_4$ 100L sample.

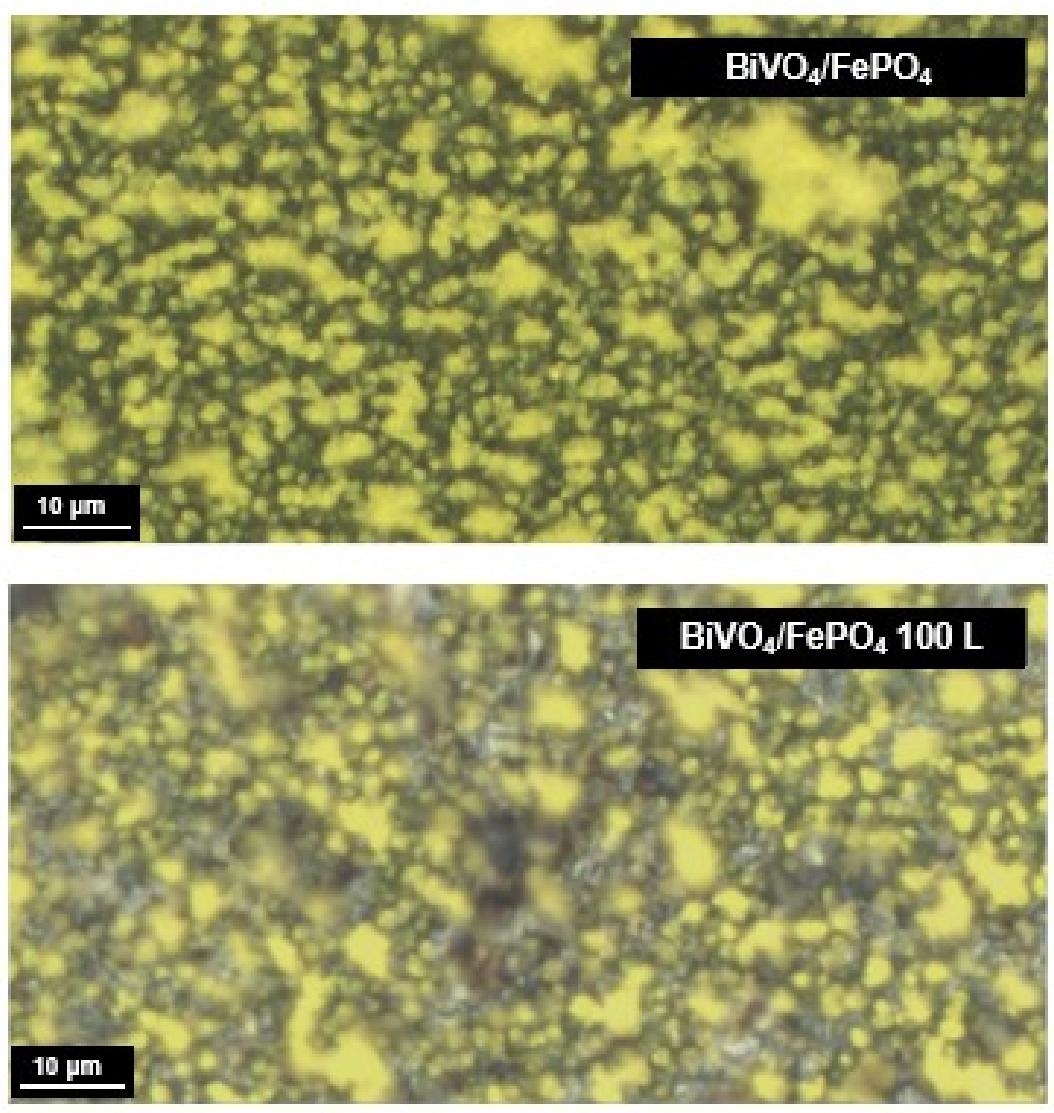

Fig. S11. White light optical micrographs (100x) of $BiVO_4/FePO_4$ and $BiVO_4/FePO_4$ 100L. A higher visual heterogeneity is apparent in $BiVO_4/FePO_4$ 100L, in line with both pre- and post-PEC SEM analysis.

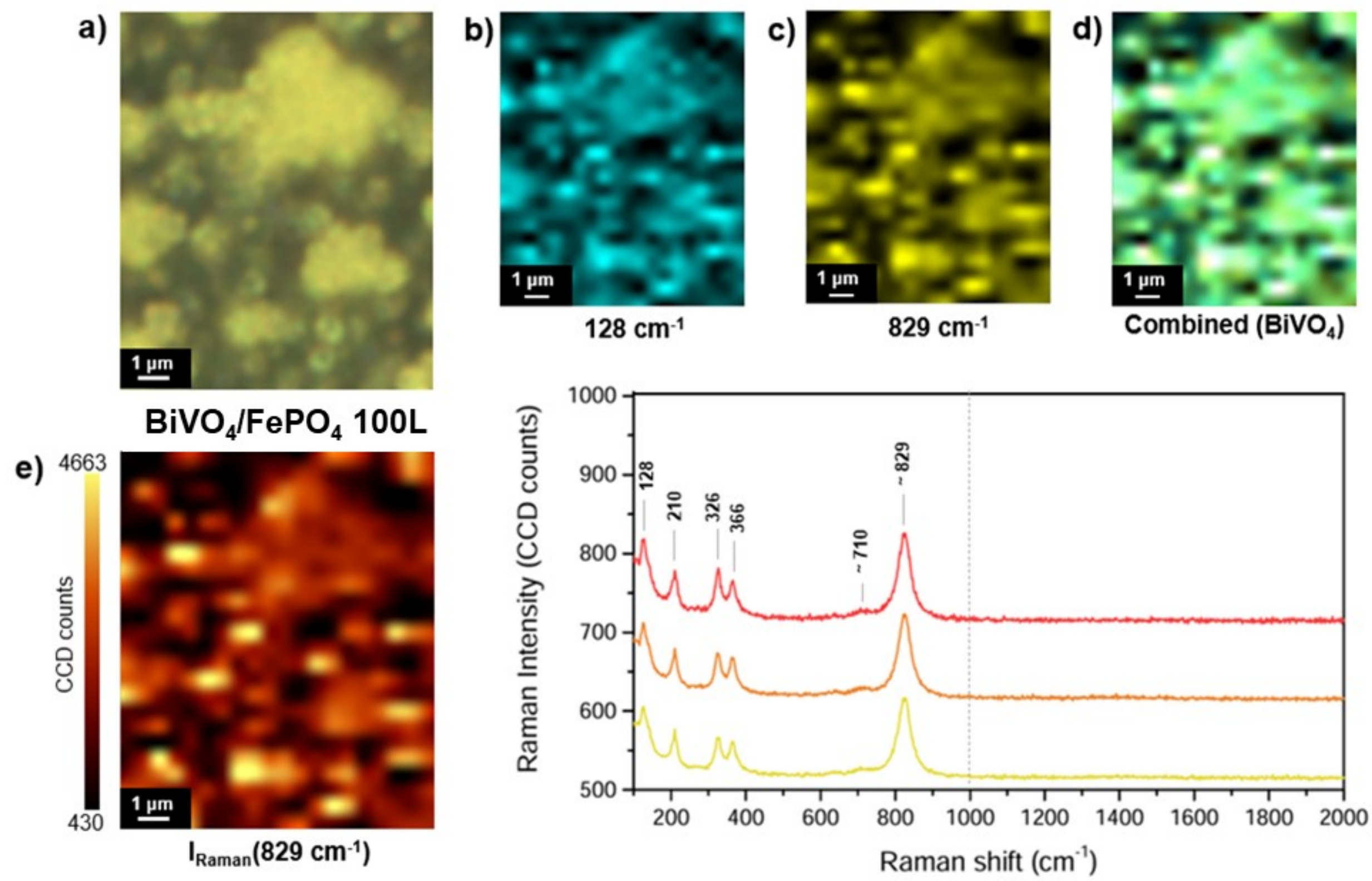

Fig. S12. Raman imaging (λ_{exc} 785 nm) of a representative area of the $BiVO_4/FePO_4$ sample after PEC measurements. a) White light optical microscopy image of the mapped area. b–d) Spatial distribution of the integrated Raman intensities centered at 128 cm^{-1}, 829 cm^{-1} and the combined 128 + 829 cm^{-1} channels, highlighting the spatial correlation between the two spectral contributions and thus the localization of $BiVO_4$. e) Heat map of the integrated Raman intensity of the V–O symmetric stretching mode across the mapped area. As shown by the CCD-count scale bar, darker regions still exhibit the characteristic $BiVO_4$ band, consistent with the expected homogeneity of crystalline $BiVO_4$ films. f) Three representative spectra extracted from the mapped sample area; no bands associated with $FePO_4$ are detected (the marker band of phosphate is expected around 1000 cm^{-1}, as indicated by the vertical dashed line). Spectra are vertically offset for clarity.

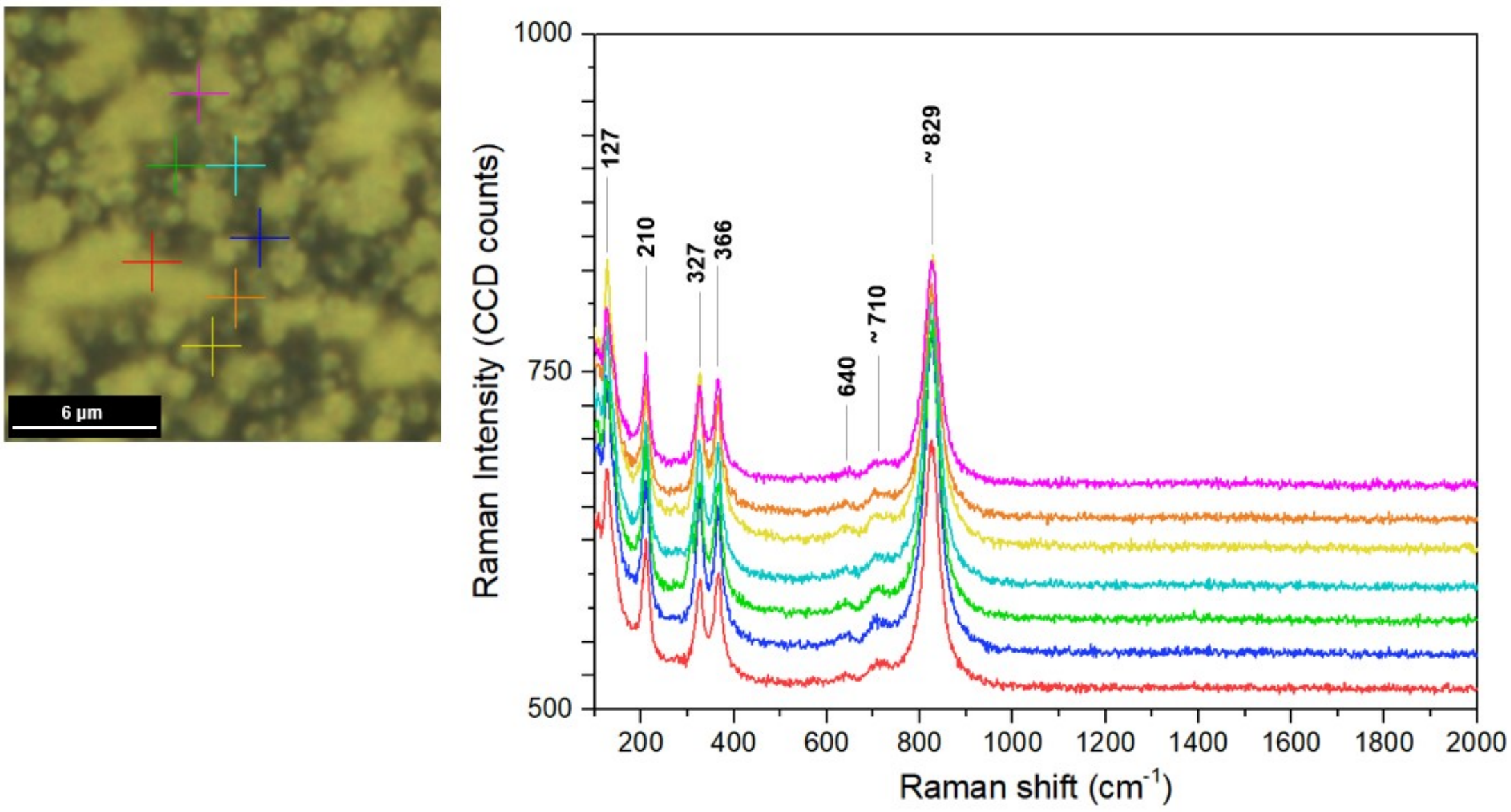

Fig. S13. Raman spectra (λ_{exc} 785 nm) extracted from 7 points across a representative area of a pristine $BiVO_4$ sample. All of them show the 640 cm^{-1} band, which is attributed to the symmetric stretch of shorter V–O bonds within the vanadate tetrahedra.

Table S3. Quantification of Bi, V, Fe, P and Ti elements before and after stability test based on high-resolution XPS spectra.

Sample	**Bi**	**V**	**Fe**	**P**	**Ti**
**Before**	1%	1%	7%	19%	72%
**after**	7%	2%	18%	35%	37%
